# Attention and learning strategies reveal distinct dimensions of psychiatric diseases

**DOI:** 10.21203/rs.3.rs-9698354/v1

**Published:** 2026-06-23

**Authors:** Warren Woodrich Pettine, Ioanna Douka, Angela Tseng, Amy Yang, Alan Anticevic, Anna R. Docherty, A. David Redish, John D. Murray, Suma Jacob

**Affiliations:** 1 Huntsman Mental Health Institute, Department of Psychiatry, University of Utah, USA; 2 Department of Psychiatry, Yale School of Medicine, USA; 3 Department of Psychiatry, University of Minnesota, USA; 4 Department of Neuroscience, University of Minnesota, USA; 5 Department of Psychological and Brain Sciences, Dartmouth, USA; 6 Department of Psychiatry, University of California, Los Angeles, USA

## Abstract

Precise measurement of individual variation in psychiatric symptoms is essential for developing scalable tools that can ultimately inform treatment development and clinical care. Individuals performing the same cognitive task often adopt qualitatively distinct strategies that may reflect differences in underlying neural processes and psychiatric symptom dimensions. Yet most cognitive paradigms implicitly assume that all individuals rely on the same cognitive strategy, attributing behavioral variability to differences along a shared computational axis. This assumption may obscure meaningful cognitive heterogeneity that cuts across traditional diagnostic categories, which themselves group phenotypically and biologically diverse individuals. Even dimensional approaches may fail to capture distinct computational profiles if they assume a common underlying cognitive process across participants.

To test this hypothesis, we combined a computationally grounded Context Generalization task involving different visual stimuli (color, shape, texture, size) with standardized self-report psychiatric questionnaires. We tested how distinct cognitive strategies relate to symptom dimensions commonly observed in autism spectrum disorder (ASD), attention-deficit/hyperactivity disorder (ADHD), obsessive-compulsive disorder (OCD), depression, and schizotypy. Participants (N=744 in session one; N=584 in session two) were recruited online, enriched for self-reported ASD diagnoses, and matched for sex at birth.

We identified qualitatively unique strategies related to goal-directed attention and short-term memory, such as a focused goal-directed strategy and a frequency-based strategy that relied on attending to all the stimulus features. The goal-directed strategy was associated with lower behavioral rigidity, while the frequency-based strategy was associated with elevated behavioral rigidity, despite showing the highest task performance. Strategy membership was substantially stable across sessions. Although transitions from the random-like strategy to the frequency-based strategy occurred less often than expected by chance, the subset of individuals who made this transition showed elevated behavioral rigidity and inattention-related symptoms consistent with the broader frequency-based profile.

These findings demonstrate that individuals vary substantially in the cognitive strategies used to solve the same task and highlight the importance of measuring inter-individual variation in computational strategy rather than relying solely on aggregate performance metrics. More broadly, our results support a framework for linking psychiatric phenotypes to interpretable patterns of attention and learning, advancing efforts toward computational phenotyping and precision psychiatry.

## Introduction

Historical approaches towards measuring cognitive domains make the assumption that every person engages in the same cognitive operations in order to perform a given task. However, individuals facing the same cognitive task can employ a wide variety of strategies (Miller, 1990; Marewski and Schooler, 2011). Despite this evidence, most experimental paradigms measure deviations from a single assumed strategy, leaving open the possibility that distinct cognitive strategy dimensions exist. Given the diversity of human behavior, such approaches may fail to fully capture meaningful heterogeneity, a critical limitation when applying these tasks to measure cognitive processes affected by psychiatric disorders.

Recent shifts in psychiatry have increasingly acknowledged that broad diagnostic categories — which often encompass phenomenological, etiological, and biologically heterogeneous syndromes — must be expanded to include dimensions that better capture variation in neurobiology and behavior (Parkes et al., 2021; Potuzak et al., 2012). However, accurate and precise measurement of psychiatric phenomenology remains a major challenge (Karvelis et al., 2023). One core issue is that individuals who exhibit psychiatric symptoms vary substantially in how they deviate from normative function and behavior. This underscores the need to develop measures that more precisely quantify variation in affected cognitive processes, such as attention and memory. Such approaches may help define more homogeneous subgroups and/or dimensional profiles that can be studied in relation to neural circuit alterations. This is a critical step toward developing precision endpoints in psychiatry, which may ultimately accelerate the development of targeted mechanism-based interventions (Anticevic and Murray, 2017; Tiego et al., 2023).

To address this gap, it is vital to combine cognitive tasks with analytical approaches capable of quantifying meaningful individual variation. For example, Collins and Frank (2012) introduced a reinforcement learning task with varying working memory (WM) demands to disentangle the contributions of distinct cognitive systems. By manipulating task conditions, such as increasing WM load, they demonstrated that computational models could separately estimate the influence of working memory and reinforcement learning (RL) processes on individual behavior (Collins and Frank, 2012). Similarly, probabilistic reinforcement learning tasks incorporating state transitions have been instrumental in elucidating individual cognitive strategies (Solomon et al., 2011). Quantifying the degree to which individuals rely on different strategies may provide insight into the boundaries of normative variation, as well as the extreme alterations associated with psychiatric symptoms (Voon et al., 2017; Bishop and Gagne, 2018). These approaches can inform dimensional behavioral profiles grounded in computational models, potentially advancing precision endpoints for psychiatry and future clinical trials.

The fundamental cognitive process of attention shows substantial variation in the general population and an even broader distribution across the clinical spectrum. Given its critical role in learning and decision-making, more precise quantification of attentional dimensionality is needed to improve our understanding of psychiatric disorders. Attention during learning shapes internal representations of the external world (Niv, 2019; Radulescu and Niv, 2019; Radulescu et al., 2021), while during decision-making, it influences feature weighting and the choices individuals make (Redish et al., 2007; Pettine et al., 2021). The ability to flexibly allocate attention to goal-relevant features is a key component of cognitive control (Sherman and Usrey, 2021) and changes across development (Raab and Hartley, 2018).

Attentional alterations observed across psychiatric disorders are well documented, including in autism spectrum disorder (ASD) (Ames and Fletcher-Watson, 2010; Dellapiazza et al., 2018), attention-deficit/hyperactivity disorder (ADHD) (Craig et al., 2016), obsessive/compulsive disorder (OCD) (Benzina et al., 2016), depression (Peckham et al., 2010; LeMoult and Gotlib, 2019), and psychosis (Keefe and Harvey, 2012). In ASD, individuals often have difficulty shifting attention from constituent parts to a broader gestalt (Kanner, 1943; American Psychiatric Association, 2013). Eye-tracking and electrophysiological studies support this observation, showing impaired disengagement from central stimuli (Sabatos-DeVito et al., 2016; Kawakubo et al., 2007) and increased focus on specific details. These impairments in attentional flexibility often reflect broader behavioral rigidity and insistence on sameness (Lage et al., 2024). Similar attentional rigidity is also observed in OCD, where individuals struggle to shift attention away from intrusive thoughts or salient stimuli (Armstrong et al., 2011). In contrast, ADHD is more commonly characterized by attentional instability and distractibility rather than perseveration. However, individuals with co-morbid ASD and ADHD exhibit more complex attentional profiles, including shared abnormalities in attention-related networks involved in cognitive control and attentional allocation (Curtin et al., 2022; Yerys et al., 2019). Collectively, these findings suggest that attentional impairments are not disorder-specific but instead reflect dimensional symptoms — such as rigidity and distractibility — that cut across traditional diagnostic boundaries. Accurately characterizing these attentional profiles may therefore help identify neurobiologically meaningful targets for future interventions.

Short-term and working memory (Baddeley, 2000; Cowan et al., 2024), which refer to the maintenance and manipulation of information over short periods (Goldman-Rakic, 1995; Jonides et al., 2008; D’Esposito and Postle, 2015), are closely intertwined with attention; therefore, performance on items presented earlier versus later can vary (Pineño and Miller, 2005). Similarly to attention, short-term and working memory are commonly affected in psychiatric disorders, including ASD, ADHD, OCD, and schizotypy (Barch and Ceaser, 2012; Benzina et al., 2016; Craig et al., 2016; Wang et al., 2017). Given the close relationship between attention and memory processes, incorporating memory-related components into cognitive tasks may provide greater sensitivity for detecting meaningful individual differences and defining more precise psychiatric phenotypes.

Together, this literature supports the view that attentional deficits are interconnected with multiple cognitive systems and shaped by diverse behavioral and biological factors. More broadly, these findings underscore the importance of conceptualizing psychiatric disorders dimensionally — an approach central to initiatives such as the Research Domain Criteria (RDoC) framework of the National Institute of Mental Health (NIMH) (of Mental Health, 2020).

To address these gaps in understanding individual variation in attention and memory strategies, we applied a novel computationally grounded Context Generalization task (Pettine et al., 2023). This approach enables the quantification of how distinct cognitive strategies relate to specific psychiatric symptom dimensions by examining covariation between task-derived parameters and well-established psychiatric questionnaires. In turn, this framework allows for the identification of computationally informed yet symptom-relevant dimensional profiles that may ultimately be linked to variations in neural targets. Such an approach has the potential to define more accurate and precise dimensions of altered behavior, refine patient stratification, and establish quantitative endpoints for measuring treatment efficacy in future clinical trials.

To investigate the association of distinct attentional and memory strategies with self-reported psychiatric symptoms, we paired the Context Generalization task with widely used, well-validated self-report measures of clinical psychiatric dimensions (Table 1). Adult participants (N = 744) were recruited online to complete two study sessions spaced four to six weeks apart (second session N = 584). For psychiatric assessment, we selected widely used and well-validated self-report measures of psychiatric symptom dimensions (Table 1). By grouping subjects according to performance, goal-directed attention and short-term memory metrics, we identified four distinct cognitive strategies: (1) High Goal-Directed (147/744), characterized by preferential attention to informative features; (2) Prototype/Exemplar (255/744), focusing on feature consistency across examples; (3) Primacy Bias (30/744), showing better generalization for early-learned stimuli; and (4) Recency Bias (70/744), showing better generalization for recently learned stimuli; the remaining subjects (240/744) varied beyond these four categories. We found that the High Goal-Directed strategy was associated with significantly lower rigidity symptoms compared to the Prototype/Exemplar strategy. Furthermore, strategy use was relatively stable across sessions, although transition patterns did not support a general shift away from less effective approaches. Together, these findings demonstrate that individual differences in attention and memory strategies are stable over time and meaningfully linked to psychiatric symptom dimensions.

## Methods

### Task structure

**Figure 1:**
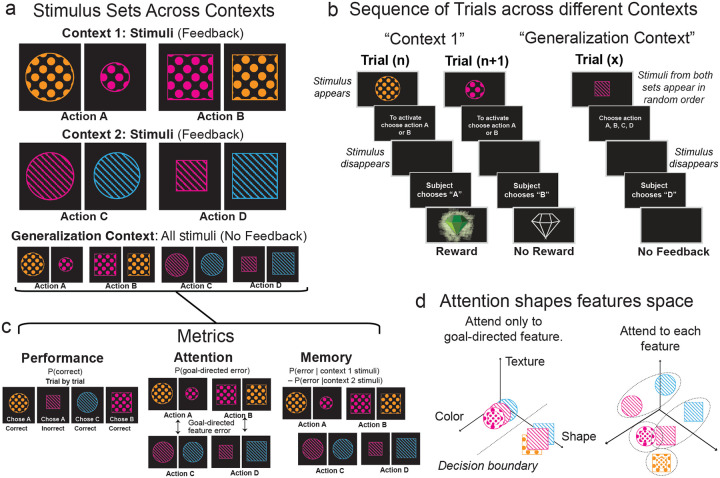
Investigating the relationship between psychological dimensions of psychiatric disorders, cognitive strategies, and shifts in cognitive strategies across time. We examined how psychiatric symptom dimensions relate to cognition by pairing commonly used questionnaires with a context generalization task that identifies qualitatively distinct approaches to attention, learning and memory. In the task, subjects viewed stimuli varying in color, shape, texture, and size, and earned points for selecting the rewarded action. The main task consisted of three explicitly signaled contexts (a) Participants first completed Context 1, viewing four stimuli (Context 1 stimuli) associated with actions *A* and *B*. Circle stimuli (large or small; magenta or orange; dotted texture) mapped to *A*, and square stimuli (large; magenta or orange; dotted texture) mapped to *B*, making shape the informative feature. In Context 2, participants viewed four new stimuli (Context 2 stimuli) and selected between actions *C* and *D*: circles (large; magenta or blue; diagonal texture) mapped to *C*, and squares (large or small; magenta or blue; diagonal texture) mapped to *D*, again making shape the informative feature. In an explicitly signaled Generalization Context, all stimuli from Context 1 and 2 were shown in random order without feedback. (b) During Contexts 1 and 2 of the task, subjects were presented with a single example stimulus and selected an action. Depending on their choice, they either received a reward or the reward was omitted, allowing them to learn the correct associations through feedback. Subjects then transitioned to a third phase (the generalization block), where they were shown stimuli from the previous contexts in random order; in this phase, no feedback was provided. Each trial represented the encounter of one stimulus and the associated action. (c) Three metrics were used to quantify behavior during the generalization block: overall performance; attention and memory. (d) Attention shapes the internal representation of stimuli. For example, attending only to a goal-directed feature collapses the internal representation onto the shape dimension, while equally attending to all features results in clear separation between stimuli.

Subjects completed a Context Generalization task designed to identify distinct cognitive strategies related to goal-directed attention, learning, and short-term memory (Pettine et al., 2023). During the task, participants viewed visual stimuli defined by color, shape, texture, and size, and selected an action in response. The correct responses earned points as a reward (Fig. 1a). There was no explicit instruction on the association between a stimulus and the actions that yielded rewards. Instead, subjects learned the associations through feedback.

The sessions started with a tutorial. During the tutorial, subjects experienced probabilistic associations between a stimulus and available actions. Thus, if they selected an action that is rewarded, they could not be certain that it was the best available action. Similarly, if a reward was omitted, they could not be certain that was the worst available action. Subjects then transitioned to the main task, where they sequentially moved between three explicitly-signaled contexts (Fig. 1b).

During the main task, the correct actions were consistently rewarded using deterministic action–reward mappings to facilitate learning (actions were deterministically mapped to a single state). In the first context, participants were presented with four stimuli (Context 1 stimuli) and two available actions (*A* and *B*). Two stimuli were associated with action *A* and the other two with action *B*. For Action *A*, the two stimuli shared shape (circles) and texture (dots), but differed in size (large and small) and color (magenta or orange). For Action *B*, the two stimuli shared shape (square), texture (dots), and size (large), but differed in color (magenta or orange). Thus, the informative dimension for actions in context 1 was shape (circle versus square). Participants completed 48 trials during context 1. Each trial represented the encounter of one stimulus and the associated action. The number of trials was chosen to ensure that they formed at least some effective representation of the states that they could use during generalization. The subjects were then explicitly signaled to enter a second context and instructed that they would encounter entirely new stimuli and new actions (*C* and *D*). They were presented with four new stimuli (Context 2 stimuli). For action *C*, the two stimuli shared the features shape (circles), size (large), and texture (diagonal lines), but differed in color (magenta or blue). For action *D*, the two stimuli shared shape (square) and texture (diagonal lines), but differed in color (magenta or blue) and size (small or large). Thus, the dimension informative for actions in context 2 was also shape, as in the first context. Similarly, as in context 1, participants completed 48 trials.

Finally, subjects entered an explicitly signaled third context, the generalization context. They were informed that all previously encountered stimuli (both from Context 1 and Context 2) and actions would be available, but that no feedback would be provided during this phase. They were also told that their compensation bonus was based on performance during the third context. Participants completed 80 trials in the generalization context.

To succeed, subjects could adopt several qualitatively distinct cognitive strategies, including: focusing on the goal-directed feature (shape) (goal-directed strategy) (Maddox and Ashby, 1993; Niv et al., 2015), memorizing each stimulus individually (exemplar strategy) (Medin and Schaffer, 1978; Estes, 1986), or categorizing each stimulus by their distance from an idealized internal-state prototype (prototype strategy) (Minda and David, 2001). Each strategy resulted in characteristic patterns of actions and errors, revealing the distinct cognitive approaches used during learning. Each attentional strategy resulted in a unique stimulus location in feature space (Fig. 1c). Importantly, locations in feature space were associated with unique patterns of actions and errors (Fig. S2) that reflected distinct cognitive strategies (Pettine et al., 2023).

### Behavioral metrics

Subject behavior was scored according to metrics for overall performance, attention, and memory (Pettine et al., 2023). All metrics were calculated during the generalization block.

#### Performance

General performance was calculated using the total rate of errors in the entire generalization context (Fig. 1d, left),

(1) $$\text{Performance}=P\left(\text{Correct}|\text{Generalization Context}\right).$$

#### Attention

If subjects preferentially attend to the goal-directed discriminative feature (shape), they exhibit a specific error type we call a “goal-directed discriminative error.” This error occurs when, due to the exclusion of previously non-discriminative feature dimensions (texture), distance between two perceptually distinct examples is reduced (Fig. 1c, left). Goal-directed discriminative error was calculated using,

(2) $$\text{Attention}=P\left(\text{Discriminative Type}|\text{Error}\right).$$

A goal-directed discriminative error rate of 33% is chance, and a score near that value may indicate random behavior. A bias towards an exemplar or mean-prototype strategy is reflected in a goal-directed discriminative error rate below 33%. Attention to prototype covariance produces a rate around 50%. A bias towards goal-directed attention is associated with a rate substantially above 50% (Pettine et al. (2023); Fig. 1d, middle).

#### Memory

The sets of stimuli were learned in sequential contexts (Fig. 1b). Thus, when subjects arrive at the generalization context, they will have just seen the Context 2 stimuli, while several minutes will have passed since they last encountered the Context 1 stimuli. A difference in memory could be indicated by a difference in performance between the Context 1 and Context 2 stimuli during the generalization context (Fig. 1d, right). We thus compared the generalization block performance of the Context 1 and Context 2 stimuli using the equation,

(3) $$\text{Memory}=P\left(\text{Error}|\text{Context}\;1\right)-P\left(\text{Error}|\text{Context}\;2\right).$$

We defined “Recency Bias” as a positive value, given that it indicates a subject does better on the more recently learned Context 2 stimuli. “Primacy Bias” was defined by a negative value, as it indicates a subject performs better on the initially learned Context 1 stimuli.

### Online task deployment

#### Pilot and replication

We first conducted a pilot study that involved 131 subjects in session 1 and 33 subjects in session 2 (sessions 1 and 2 were 4–6 weeks apart). Using data from the pilot study, we developed hypotheses and pre-registered our analysis. That preregistration was made on April 27, 2023, and can be found at OSF ([details omitted for anonymized review]). We then conducted a full study with 744 subjects during session 1 and 584 during session 2. Although we performed and reported all the analyses from preregistration, given the larger sample size in the replication study, we were also able to perform more sophisticated analyses in session 2 that were not preregistered.

#### Subjects

We recruited subjects through the online platform Prolific (Palan and Schitter, 2018) for both the pilot and replication samples. To participate, subjects were required to speak English as a first language and be located in the US, UK, Canada, Ireland, Australia, or New Zealand. These requirements were in place to ensure the validity of the English-language questionnaires. Subjects were also required to be over 18 years old, and recruitment was balanced for self-reported sex. With our interest in context generalization differences in those with Autism Spectrum Disorder (ASD) symptoms, we also recruited 50% of participants who reported having a formal diagnosis of ASD.

#### Task implementation and recruitment

This study was approved by the University of Minnesota Institutional Review Board (IRB). All participants consented to an IRB-approved online informed consent document and were paid for their participation. After consenting to participate, they answered a few basic demographic questions (age, level of education, gender, sex; Supplementary Table **??**), and completed several psychiatric symptom questionnaires (Table 1). The Conners inconsistency score measures the degree to which a subject’s responses vary when they answer similar questions. The higher the score, the larger the deviation (Conners, 2008). Subjects who passed the attention check questions and achieved a Conners inconsistency score below 8 were then invited to participate in the first task session. Four to six weeks later, all subjects were invited to participate in a second session (Fig. 1e). At the end of a task session, subjects were asked to rate the difficulty of the task, provide a verbal description of their strategy, and then choose an option that they felt best described their strategy. The task structure and the use of stimuli were the same for both session 1 and session 2, although the cover story was changed. We implemented the questionnaires and tasks as a web app using the Django framework and hosted them online through Microsoft Azure.

#### Human subject compensation

Human subjects received $2 for completing the tutorial (whether passing or not), $4 for completing the task, and $4 for achieving a performance level above 60% during the generalization context. For the second session, subjects received $2.50 for completing the game and a $3 bonus for exceeding the 60% performance level.

### Identification of cognitive groups

We identified cognitive strategies by first clustering subjects according to their behavioral metrics and then fitting models to their patterns of errors.

### Clustering subjects according to behavioral metrics

We clustered subjects according to their scores for performance, attention, and memory using agglomerative clustering with an average linkage function from the Scikit-learn package (Pedregosa et al., 2011). To determine the number of clusters, we inspected the dendrogram to identify branch points at which qualitatively distinct cognitive strategies were clearly separated. This approach allowed us to determine where meaningful strategy divisions emerged and to define clusters reflecting these distinct cognitive patterns.

### Confusion matrices

We created confusion matrices of each cluster’s pooled errors. Rows in the confusion matrix indicate the presented examples, and the columns indicate selected actions on error trials. The diagonal of the matrix is empty, as those are for actions associated with the most-rewarded option.

### Attention models

We formulated idealized models to characterize how participants attend to specific stimulus features, assuming they were operating under distinct cognitive strategies. Predictions were generated based on attentional patterns at the beginning of the generalization context, only after participants had learned all stimulus-response associations, but before those associations were presented together. Distinct forms of attention, learning, and memory can be inferred by examining predicted patterns of errors in the resulting confusion matrix (Pettine et al., 2023). For example, a uniformly distributed error pattern may indicate random responding, whereas memorizing each stimulus individually, without generalization, typically produces errors that vary as a function of feature distance between stimuli. In contrast, goal-directed attention leads to errors concentrated on stimuli that share the same task-relevant discriminative feature, such as shape. When participants rely on a prototype-based strategy (stimuli are categorized by their distance from an internal-state prototype/attend to all features), their errors are structured around the mean and covariance of that prototype representation. Similarly, an exemplar-based strategy (stimuli are categorized based on past state examples/attend to all features) yields an error pattern that reflects the distribution of individual stimuli previously encountered. Memory-related influences can also shape response patterns: a primacy bias manifests as stronger performance on early-learned stimuli and more errors on recently encountered ones, while a recency bias produces the opposite effect. These memory effects can also occur with high-goal directed error patterns, but those cases may be considered a subset of the high-goal directed attentional strategy (Fig. S2).

### Fitting confusion matrices

We employed Bayesian linear regression to fit the idealized attentional model confusion matrices to the observed confusion matrices and conducted subsequent model comparisons. Each confusion matrix was vectorized, and predictor variables were assigned half-normal priors on their coefficients. A half-normal prior was also applied to the variance term, while the intercept term followed a standard normal prior. The models were implemented using the Bambi Python package (Capretto et al., 2022) and estimated by Monte Carlo sampling of the Markov chain with the following settings: tune=2000, draws=2000, init=advi, and target accept=0.9. We evaluated the quality of the fits through the trace of each distribution and $\hat{r}$, as well as inspecting the posterior predictive distribution.

#### Model comparison

To evaluate the contribution of each idealized attentional predictor, we fit each attentional model separately, along with the intercept term, to each cluster’s observed pattern of errors (Figs. S4 and S10). Both prototype and exemplar/mean-based attentional models were included as predictors in their respective fits. We also fit a null model that included only the intercept and *σ* distributions. The quality of fit was determined using Pareto smoothed importance sampling leave-one-out cross-validation (Vehtari et al., 2017), as implemented by the Arviz Python package (Kumar et al., 2019).

### Grouping by cognitive strategy

We grouped clusters according to their best fitting cognitive strategy model (Figs. S3 and S9). Thus, if two clusters shared the same cognitive strategy, they were included in the same group.

### Session performance comparison analysis

#### Strategy shift and consistency

To analyze the relationship between strategy choices across the two sessions, we performed a Chi-square test of independence. This statistical test is used to determine whether there is a significant association between two categorical variables, in this case, the strategy choices in Session 1 and Session 2.

The test was conducted on a 5×5 contingency table, where rows represented the strategies used in Session 1 and columns represented the strategies used in Session 2. The five strategy categories were High Goal-Directed, Other, Primacy Bias, Prototype/Exemplar, and Recency Bias. The Chi-square test compares the observed frequencies in each cell of the contingency table to the frequencies that would be expected if there were no association between the variables. The test statistic is calculated using the equation,

(4) $$\chi^{2}=\sum \frac{{\left(O-E\right)}^{2}}{E},$$

where O is the observed frequency and E is the expected frequency for each cell. The null hypothesis for this test is that there is no association between strategy choices in Session 1 and Session 2, while the alternative hypothesis is that there is an association. We also calculated Cramer’s V, a measure of effect size for the Chi-square test of independence. Cramer’s V ranges from 0 to 1, with values closer to 1 indicating a stronger association. It is calculated using,

(5) $$V=\sqrt{\frac{\chi^{2}}{n(k-1)}},$$

where n is the total number of observations and k is the smaller of the number of rows or columns in the contingency table. To identify specific strategy transitions that significantly deviated from expected frequencies, we calculated standardized residuals for each cell in the contingency table. Standardized residuals with absolute values greater than 1.96 were considered statistically significant at the p<0.05 level. All statistical analyses were performed using Python with the scipy and numpy libraries.

#### Behavioral shift metrics

We computed the following metrics for each subject to assess the shift in behavior across sessions:

(6) $$\text{Performance Error Differenc}e=\left(\text{Performance}|\text{Session}\;1\right)-\left(\text{Performance}|\text{Session}\;2\right);$$

(7) $$\text{Attention Difference}=P\left(\text{Attention Score}|\text{Session}\;1\right)-P\left(\text{Attention Score}|\text{Session}\;2\right);$$

(8) $$\text{Memory Difference}=P\left(\text{Memory Score}|\text{Session}\;1\right)-P\left(\text{Memory Score}|\text{Session}\;2\right).$$

We then used K-means to cluster these metrics according to the preregistered four clusters, as well as an exploratory six clusters.

### Psychiatric symptoms-behavior analyses

#### Questionnaires and scoring

Subjects answered questions from several commonly used psychiatric symptoms questionnaires and were scored according to established criteria (Table 1). There were four questions to check if subjects were paying attention. Incorrect responses to at least two attention check questions resulted in rejection of the subject’s response.

#### Symptom-group analyses

We first grouped subjects according to cognitive strategies. Then for each subscale of the psychiatric symptoms questionnaires we performed a one-way ANOVA to determine if the score differed across the cognitive strategies, with a significance threshold of P<0.05. Given the high degree of positive dependence across subscales, we used the Benjamini-Hochberg procedure to correct the ANOVA p-values (Benjamini and Hochberg, 1995).

#### Regression of group membership

We z-scored all subscale responses across subjects and performed a series of multinomial logistic regressions to identify predictors of cognitive strategy use. All models controlled for demographic variables including age, sex assigned at birth, reported gender, geographic region, education level, and caffeine use within three hours. The predictor sets for each model were:

$$X_{\text{ReportedDiagnoses}}=(\text{Addiction, Depression, OCD, ADHD, ASD, Schizophrenia, Schizotypy,}\;1)$$

$${\text{X}}_{\text{QuestionnaireCutoffs}}=\left(\text{BAPQ},{\text{Conners ADHD Index, CAPE, PHQ9}}_{\text{mild}},\text{PHQ9}9_{\text{moderate}},\text{PHQ9}9_{\text{severe}–\text{very severe}},1\right)$$

$${\text{X}}_{\text{QuestionnaireScores}}=(\text{BAPQ, Conners ADHD Index, CAPE, PHQ9, RBQ2a, OLIFE-s},1)$$

$${\text{X}}_{\text{QuestionnaireSubscales}}=\left({\text{BAPQ}}_{\text{Aloofness}},{\text{BAPQ}}_{\text{PragmaticLanguage}},{\text{BAPQ}}_{\text{Rigidity}},{\text{Conners}}_{\text{DSM-H}/\text{I}},\right.\;{\text{Conners}}_{\text{Inattention/Memory}},{\text{ConnerS}}_{\text{DSM-Inattention}},{\text{Conners}}_{\text{Hyperactivity/Restlessness}},\;{\text{Conners}}_{\text{DSM-Impulsivity/EL}},{\text{Conners}}_{\text{Self-Concept}},{\text{ASRS}}_{\text{Inattention}},{\text{ASRS}}_{\text{Hyperactivity/Impulsivity}},\;{\text{OCIR}}_{\text{Washing}},{\text{OCIR}}_{\text{Checking}},{\text{OCIR}}_{\text{Neutralizing}},{\text{OCIR}}_{\text{Ordering}},\;{\text{OCIR}}_{\text{Hoarding}},{\text{OCIR}}_{\text{Obsessing}},{\text{CAPE}}_{\text{Positive}},{\text{CAPE}}_{\text{Negative}},\;{\text{RBQ2a}}_{\text{RepetitiveMotor}},{\text{RBQ2a}}_{\text{InsistenceOnSameness}},{\text{RBQ2a}}_{\text{Other}},\;{\text{OLIFE-s}}_{\text{CognitiveDisorganization}},{\text{OLIFE-s}}_{\text{UnusualExperiences}},{\text{OLIFE-s}}_{\text{IntrovertedAnhedonia}},1)$$

### Linear discriminant analysis of individual questions

We used linear discriminant analysis (LDA) to determine which specific questions were driving the differences between the cognitive strategy groups. To do this, we first filter the individual questions by their significance in distinguishing between the clusters. This was done with a one-way ANOVA, including only the questions with p<0.01. Of the questions that passed filtering, we performed leave-one-out cross-validated (LOO-CV) LDA to identify dimensions significant for distinguishing between the clusters. To generate a control distribution, we performed the LOO-CV LDA 10 times with shuffled cluster labels. We then averaged the LOO-CV of the true distribution and determined significance by measuring the proportion of control values for which the true distribution was greater. We averaged the scalings for each question for each discriminant across cross-validated runs. Those questions whose mean scaling absolute value > 1 were considered significant.

## Results

We investigated the association between dimensions of psychiatric disorders and cognition by pairing commonly used questionnaires (Table 1) with a context generalization task that identifies qualitatively different approaches to attention and memory (Pettine et al., 2023). A sample of subjects enriched for self-reported formal diagnosis of ASD was recruited online to answer questions and complete two sessions (session 1 N=744, session 2 N=584) separated by 4–6 weeks. In the context generalization task, participants responded to visual stimuli defined by color, shape, texture, and size, earning points for selecting the rewarded action. To successfully learn the task, they could employ several qualitatively distinct cognitive strategies. Focusing on the goal-directed feature (shape) was reflected in the goal-directed strategy. Focusing on features based on an internal composite representation or consistency across past examples indicated a prototype/exemplar strategy. Attending more to features first encountered reflected a primacy bias strategy, whereas attending more to features most recently encountered reflected a recency bias strategy. Subjects who did not fit any of these patterns were classified as following the ‘other’ strategy. Each strategy produced distinct patterns of actions and errors, providing insight into the attentional and memory processes used to solve the task. These approaches were associated with psychological dimensions related to rigidity and inattention observed across disorders, including ASD, ADHD, OCD, and psychosis.

Cognitive strategies were substantially stable across sessions; among participants who changed strategies, session-two Prototype/Exemplar membership was associated with symptom profiles similar to those observed in participants assigned to that strategy more generally. This underscores the importance of quantitatively precise mapping of cognitive strategies, which may correspond to distinct phenotypic endpoints associated with psychiatric symptom dimensions.

### Cognitive strategies occupy distinct locations in the task metric space

**Figure 3:**
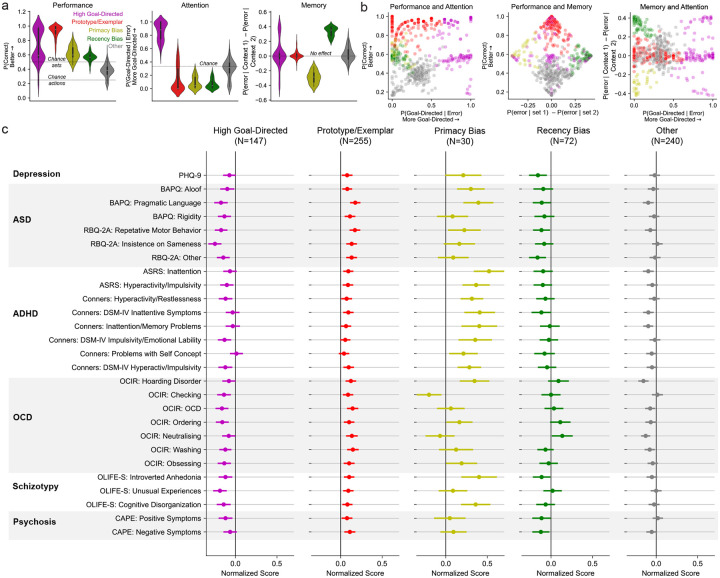
Cognitive strategies identified by using metrics for performance, attention and memory exhibit contrasting patterns in dimensions of psychiatric disorders (Session 1). (a) Subjects grouped according to cognitive strategy differed in their generalization context metric scores. (b) Cognitive strategies were identified as clusters within the task-metric space. (c) Questionnaire responses were significantly different between cognitive strategies across several subscales during Session 1. Subscale scores were normalized by z-scoring across all subjects. Shown are the mean and standard error of the mean.

We identified cognitive strategies by clustering participants who completed both sessions according to behavior during the generalization context of session 1 using task metrics for performance, attention, and memory (Fig. 3). The cognitive models were then fit to pooled patterns of errors (Figs. S2, S4), and clusters were grouped according to their best-fitting cognitive model (Fig. S3). We found that the cognitive strategies were well separated in the task metric space.

The Prototype/Exemplar group (N=201) exhibited the highest overall performance. Although this strategy involved over-learning initially non-informative features, those same features became essential during generalization, resulting in superior performance. In contrast, subjects in the Other strategy group (participants whose behavior did not conform to a distinct strategy) (N=180) demonstrated the poorest performance, with error patterns resembling randomness, suggesting that they had not successfully learned the task structure. The performance distribution of the High Goal-Directed cognitive strategy (N=116) was bimodal and its memory distribution was trimodal. This heterogeneity reflected different approaches within the group: some individuals reduced their internal representation to a single goal-relevant feature dimension (see Pettine et al., 2023), while others developed richer representations but remained biased toward the goal-directed feature. Finally, the Primacy Bias (N=24) and Recency Bias (N=63) groups showed similar performance and attention metrics, but diverged sharply on memory-related metrics, occupying opposite ends of the memory dimension.

In general, by clustering based on task-derived metrics and grouping clusters by cognitive model fit, we identified interpretable and qualitatively distinct subgroups of participants, each reflecting a unique strategy to integrate attention, memory, and learning. Together, these error profiles offer a powerful means of distinguishing between distinct cognitive strategies and uncovering the underlying attentional and memory processes that shape behavior, which is critical to guide the development of cognitive endpoints for ‘precision psychiatry’.

### Psychological dimensions of psychiatric disorders vary according to cognitive strategy

Having grouped the participants according to qualitatively distinct cognitive strategies, we aimed to characterize their psychiatric symptom profiles. We next examined whether psychiatric symptom dimensions differed across cognitive strategies. Such subgroups could inform more precise treatment interventions and guide future research efforts. Based on data from the pilot sample, we developed and preregistered a set of hypotheses and analytical plans (see Appendix A for additional preregistered analyses).

### High Goal-Directed strategy is associated with reduced rigidity symptoms relative to the Prototype/Exemplar strategy.

We first performed a preregistered ANOVA to examine differences in scores on BAPQ, Conners Index, and PHQ-9 across four cognitive strategy groups in subjects who completed both sessions: High Goal-Directed, Prototype/Exemplar, Primacy Bias, and Recency Bias. Among these measures, only the BAPQ showed significant differences across groups (p<0.05), with the Prototype/Exemplar group exhibiting higher overall BAPQ scores. We then performed pairwise Student’s t-tests comparing the High Goal-Directed group to the others. These tests revealed that the High Goal-Directed group had significantly lower BAPQ scores than the Prototype/Exemplar and Primacy Bias groups (p<0.05). Subsequently, we ran a preregistered ANOVA on relevant subscales, specifically: BAPQ Rigidity, Conners DSM-IV Inattention, and Conners Inattention/Memory Problems. Of these subscales, only BAPQ Rigidity showed significant group differences. Pairwise comparisons of the High Goal-Directed group’s BAPQ Rigidity scores with those of the other groups revealed that only the Prototype/Exemplar group exhibited significantly higher rigidity scores (p<0.05).

The High Goal-Directed and Prototype/Exemplar strategies represent contrasting cognitive profiles: the former involves selective attention to task-relevant features, while the latter entails broader attention to frequently encountered features. Given significant differences between these groups in several preregistered analyses, we conducted an exploratory comparison of their scores across all questionnaire subscales among participants who completed both sessions. Using Student’s t-tests (p<0.05), the Prototype/Exemplar group showed significantly higher scores on the BAPQ Pragmatic Language and Rigidity subscales; RBQ-2A subscales for Repetitive Motor Behavior, Insistence on Sameness, and Other; Conners DSM-IV Hyperactivity/Impulsivity; multiple OCIR subscales including Hoarding Disorder, Checking, OCD, Ordering, Neutralizing, Washing, and Obsessing; and the OLIFE-S subscales for Introvertive Anhedonia, Unusual Experiences, and Cognitive Disorganization. After correcting for multiple comparisons using the Benjamini–Hochberg procedure, significant group differences remained for BAPQ Pragmatic Language and Rigidity, RBQ-2A Repetitive Motor Behavior, Insistence on Sameness, and Other, OCIR OCD, OCIR Obsessing, Ordering, and Washing, and OLIFE-S Unusual Experiences and OLIFE-S Cognitive Disorganization (Fig. 3c).

Although our preregistered hypothesis that the High Goal-Directed group would exhibit reduced symptoms of depression and ADHD-related inattention was not supported in the replication sample, the findings consistently linked the Prototype/Exemplar strategy to elevated rigidity-related symptom dimensions. Together, these results suggest that broader, less selective attentional strategies are associated with increased behavioral rigidity across psychiatric symptom domains.

### Broader autistic phenotype scores were better than reported ASD diagnosis at predicting the use of goal-directed discriminative attention.

To better understand whether differences in the use of goal-directed discriminative attention were more strongly associated with self-reported ASD diagnosis or self-reported autistic phenotypes, using the BAPQ questionnaire, we conducted a preregistered Bayesian regression analysis. This model used two predictors: formal self-reported diagnosis of ASD and whether participants scored above the clinical cutoff on the BAPQ. The BAPQ cutoff predictor showed consistently larger effects than formal ASD diagnosis (>97.4%) (Fig. S5B). These findings support our preregistered hypothesis that self-reported autistic phenotypes are more predictive of goal-directed attention use than diagnostic status alone.

### Learning order biases show exploratory associations with psychological dimensions of psychiatric disorders.

Primacy and recency effects are classically associated with the preferential encoding of early versus recently encountered information. Primacy effects are generally attributed to greater attentional focus and cognitive resources allocated to early items, whereas recency effects are associated with the benefits of working memory for items encountered later (Kahana, 2020). Following our preregistered memory metric, we defined Primacy Bias as better generalization for initially learned stimuli and Recency Bias as better generalization for more recently learned stimuli.

Although the number of pilot subjects in the Primacy Bias (N=6) and Recency Bias (N=12) groups was limited, we preregistered hypotheses based on observed group differences. In the replication sample, these preregistered Primacy-versus-Recency hypotheses were not supported: the groups did not differ significantly on the BAPQ (p>0.05) or on the Conners inattention subscales (p>0.05). We therefore conducted an exploratory analysis across all subscales. Before correction for multiple comparisons, the Recency Bias group scored significantly lower than the Primacy Bias group on the BAPQ Pragmatic Language subscale; the ASRS subscales for Inattention and Hyperactivity/Impulsivity; the Conners subscales for Hyperactivity/Restlessness and DSM-IV Inattentive Symptoms; and the OLIFE-S subscales for Introvertive Anhedonia and Cognitive Disorganization. However, none of these exploratory findings survived Benjamini–Hochberg correction.

### Significant relationship in cognitive strategies across sessions

We fitted the cognitive strategy models to each subject’s behavior during the second session and tracked transitions in strategy use across sessions. A Chi-square test of independence showed that strategy membership was significantly related across sessions (Chi-square statistic = 128.4238, p<0.05, Cramer’s V=0.2345). Subjects were significantly more likely to maintain their original strategy than expected by chance for the High Goal-Directed, Prototype/Exemplar, Primacy Bias, and Other groups (z ≥ 1.96, p<0.05). Recency Bias did not show significant consistency across sessions (—z— < 1.96, p>0.05).

When examining transitions between different strategies, significantly more subjects transitioned from Recency Bias to Primacy Bias than expected by chance (z ≥ 1.96, p<0.05). In contrast, transitions from Other to Prototype/Exemplar and from Prototype/Exemplar to Other occurred significantly less often than expected by chance (z < −1.96, p<0.05).

Together, these findings indicate that cognitive strategies were largely stable over time. The transition results did not support a general pattern of movement from less effective to more effective strategies with repeated task exposure. Instead, the dominant pattern was stability, with one overrepresented transition from Recency Bias to Primacy Bias.

### Psychological dimensions across disorder subscales relate to cognitive strategies across sessions

To better understand how psychological dimensions relate to strategy consistency over time, we analyzed the symptom profiles of subjects who either maintained or shifted cognitive strategies across sessions. Due to the limited number of subjects in the Primacy and Recency Bias groups, analyses were restricted to participants who initially used the High Goal-Directed, Prototype/Exemplar, or Other strategies. For each group, we performed one-way ANOVAs on questionnaire subscales, comparing those who maintained their original strategy to those who transitioned to one of the other two main strategies.

Before correcting for multiple comparisons, we found that transitions from the High Goal-Directed group were associated with significantly elevated RBQ-2A Repetitive Motor Behavior scores (p<0.05). Transitions from the Prototype/Exemplar group were associated with higher scores on the BAPQ Aloofness scale, and Conners subscales for Inattention, Inattention/Memory Problems, and Hyperactivity/Impulsivity (p<0.05). Transitions from the Other group were significant across nearly all subscales, including BAPQ, RBQ-2A, ASRS, Conners, OCIR (Hoarding, OCD, Ordering), and OLIFE-S Cognitive Disorganization (p<0.05).

After applying the Benjamini–Hochberg correction, only the transitions from the Other group remained significant (p<0.05), with the exception of the OCIR subscales for OCD and Ordering. In all groups, a higher severity of psychiatric symptoms was observed in subjects who either transitioned to or remained in the Prototype/Exemplar strategy.

These findings highlight a robust association between the Prototype/Exemplar strategy and elevated self-reported psychopathology over time.

### Shifts in task metrics across sessions were associated with ASD and ADHD symptoms

Due to the limited number of participants who completed session 2 in the pilot sample (N=33), preregistered analyses focused on changes between sessions in individual performance, attention, and memory metrics rather than on clustering-based cognitive strategies. Initial analyses using a predefined four-cluster solution revealed that only behavioral rigidity differed significantly between clusters.

Exploratory analyses in the larger replication sample (N=584), however, indicated that a six-cluster solution better captured variability in longitudinal task metrics (Fig. S8). Within this expanded sample, reduced performance in session 2 was associated with elevated scores across several psychiatric dimensions, including Conners Hyperactivity/Restlessness, BAPQ Aloofness, Rigidity, and Pragmatic Language, as well as RBQ-2A Repetitive Motor Behavior, Insistence on Sameness, and Other symptoms. Together, these findings support the relationship between longitudinal shifts in cognitive strategy-related task metrics and dimensions of rigidity, inattentiveness, and hyperactivity-related psychopathology.

## Discussion

In this study, we examined how attention and learning strategies relate to dimensional measures of psychiatric symptoms by combining the Context Generalization task with standardized self-report questionnaires. We found that individual cognitive strategies were significantly associated with multiple symptom dimensions and showed substantial stability across sessions. These findings indicate that precise quantification and characterization of dimensional attentional profiles can meaningfully support the identification of precision cognitive endpoints. Together, these findings support strategy-based transdiagnostic approaches as a promising framework for characterizing clinically meaningful cognitive variation and developing precision behavioral endpoints.

### A paradigm for investigating attention and learning strategies

Prior work demonstrated that qualitatively distinct attention and learning strategies can be reliably identified using a brief Context Generalization task (Pettine et al., 2023). Although that study proposed potential cognitive mechanisms through computational modeling, it did not assess the psychological dimensions that might distinguish these strategy groups. In the present study, we show that these strategies are significantly associated with specific psychiatric symptom dimensions, particularly those related to behavioral rigidity. These findings strengthen the utility of the paradigm as a tool for characterizing meaningful individual differences in cognitive functioning. Moreover, when paired with standardized psychiatric instruments, this approach offers a powerful means to identify symptom dimensions that extend beyond traditional diagnostic categories.

### Superior performance of subjects with elevated psychiatric symptoms.

We observed that the highest-performing group (Prototype/Exemplar) showed elevated scores on psychiatric dimensions related to behavioral rigidity. These findings contrast with much of the psychometric literature, where elevated psychiatric symptoms are often associated with poorer cognitive task performance (Happé et al., 2006; Bos et al., 2019; Keefe and Harvey, 2012; Kalanthroff et al., 2016; Ramos et al., 2020). Importantly, many conventional cognitive paradigms reward strong goal-directed attention and efficient distractor filtering. In contrast, the Context Generalization task was deliberately designed to capture situations in which excessive reliance on goal-directed attention can impair generalization. In this task, initially irrelevant features later become behaviorally informative, favoring broader attentional allocation and integration across stimulus dimensions. This distinction may help explain why individuals exhibiting greater rigidity-related symptom dimensions showed superior task performance. Our findings are consistent with prior work showing that autistic individuals can outperform neurotypical individuals on specific visual attention tasks, potentially due to atypical attentional allocation, heightened focus on details, and difficulty disengaging from salient features (Kaldy et al., 2016). Related eye-tracking and neuroimaging studies suggest that enhanced attention toward highly salient or personally relevant features may support domain-specific perceptual expertise in some autistic individuals (Bos et al., 2019). Together, these findings highlight that distinct cognitive strategies may confer advantages depending on task demands and environmental structure. Recognizing both the strengths and challenges associated with neurodivergent cognition is important for developing cognitive paradigms that better capture real-world variability (Pellicano and den Houting, 2022; Baron-Cohen et al., 2009). More broadly, these findings support the idea that rigidity-related attentional profiles should not be conceptualized solely as deficits, but also as reflecting alternative modes of information processing that may prove advantageous in specific clinical contexts.

### High Goal-Directed attention versus Prototype/Exemplar

The High Goal-Directed and Prototype/Exemplar groups were the largest strategy groups in our sample and demonstrated the most robust and reproducible findings across analyses. Importantly, these qualitatively distinct attentional allocation strategies were associated with markedly different rigidity scores across multiple psychiatric symptom dimensions. Specifically, the High Goal-Directed strategy emphasized selective attention toward task-relevant features, whereas the Prototype/Exemplar strategy reflected broader attention across frequently encountered stimulus dimensions. These findings suggest that differences in how individuals allocate attention may represent a transdiagnostic cognitive dimension closely linked to behavioral rigidity.

This interpretation is consistent with prior work showing that autistic individuals often exhibit heightened attention to details and difficulty disengaging from salient features, patterns commonly described as “missing the forest for the trees” (Happé and Frith, 2006). Similar forms of attentional rigidity have also been described in OCD, where individuals may persistently allocate attention toward intrusive or highly salient stimuli (Armstrong et al., 2011; Gruner and Pittenger, 2017). In contrast, ADHD is more commonly associated with attentional instability and distractibility rather than persistent over-allocation toward specific features (Taurines et al., 2012; Kern et al., 2015; Antshel et al., 2016). Together, these findings support the idea that rigidity-related attentional phenotypes cut across traditional diagnostic categories and may reflect shared underlying cognitive mechanisms.

Notably, the Prototype/Exemplar group exhibited elevated rigidity-related symptoms across multiple questionnaire domains, including OCIR dimensions associated with compulsive behavior. This suggests that broader attentional allocation strategies may relate not only to ASD-associated rigidity, but also to transdiagnostic dimensions relevant to OCD and related conditions. Collectively, our findings support a framework in which distinct attentional allocation strategies map onto clinically meaningful dimensions of rigidity, offering a potential pathway toward identifying biologically and cognitively informed subgroups for future precision psychiatry approaches.

### Primacy Bias versus Recency Bias

While most participants showed similar generalization performance across the two stimulus sets, a small subset demonstrated clear asymmetries. Individuals in the “Primacy Bias” group performed better on the first set, whereas those in the “Recency Bias” group performed better on the second. These groups also exhibited opposite patterns on several ADHD-related subscales, particularly hyperactivity/impulsivity and inattention, with the Primacy Bias group showing higher symptom levels. Although the Context Generalization paradigm differs from classical list-learning tasks, similar attention and working-memory systems likely contribute to these primacy and recency patterns (Kahana, 2020).

A substantial body of evidence links working-memory (WM) deficits to neurodevelopmental conditions, including ADHD, where WM impairments represent a core cognitive difficulty (Duan et al., 2021). Individuals with ADHD often struggle when required to maintain and reorder stimuli, or when integrating newly presented information with existing WM representations (Kasper et al., 2012; Martinussen et al., 2005). Impaired coupling between attention and WM has also been consistently reported. Notably, WM impairments rarely operate in isolation but interact with other processes, including attention. For example, although WM training can improve task-specific performance, its effects on broader daily functioning tend to be limited (Ortega et al., 2020).

Computational work further highlights the importance of modeling WM contributions: even in simple instrumental learning tasks, capacity limitations can account for behavioral variability that might otherwise be attributed to reinforcement learning (Collins and Frank, 2012). Similar findings in schizophrenia research demonstrate that learning deficits initially interpreted as RL impairments can, in fact, reflect underlying WM dysfunction.

Although preliminary, our findings suggest that WM-related influences on attentional and learning processes may contribute to psychological dimensions such as inattention and impulsivity—dimensions that carry transdiagnostic relevance across neurodevelopmental and psychiatric disorders. Identifying subgroups characterized by specific cognitive profiles may be essential to develop more targeted, individualized interventions. This represents a promising direction for future work.

### Test Reliability

A common challenge in computational psychiatry is ensuring the stability of behavioral measurements across repeated task administrations (Zorowitz and Niv, 2022; Karvelis et al., 2023). If behavioral metrics or model-derived parameters are to function as clinical assays, they must demonstrate reliable test–retest properties (Pettine et al., 2024). Variability across sessions can arise from several sources, including shifts in affective state, changes in underlying symptomatology, medication effects, or practice-related changes that accompany repeated task exposure (Zorowitz and Niv, 2022; Karvelis et al., 2023).

The Context Generalization task offers strong construct validity through its foundation in forward-model simulations and its grounding in mechanistic and artificial neural network theory (Pettine et al., 2023). At the same time, the task is designed to reveal how participants allocate attention and use memory when previously irrelevant features become informative during generalization. Because participants complete the task twice, the second session provides an opportunity to ask whether these cognitive strategies reflect stable individual differences or flexible responses to repeated task experience.

Our results support substantial stability in strategy membership across sessions. Strategy use in session 1 was significantly related to strategy use in session 2, and subjects classified as High Goal-Directed, Prototype/Exemplar, Primacy Bias, or Other were more likely to remain in the same strategy group than expected by chance. In contrast, Recency Bias did not show significant within-strategy stability. These findings suggest that several strategy classes capture reproducible individual differences in how participants solve the task, rather than purely transient behavioral states.

The transition results did not support a general shift from less effective to more effective strategies with repeated task exposure. Although some participants transitioned from Other to Prototype/Exemplar, this transition occurred less often than expected given the marginal distribution of strategy membership across sessions. Similarly, transitions from Prototype/Exemplar to Other were also underrepresented. The clearest overrepresented transition between different strategies was from Recency Bias to Primacy Bias. Thus, repeated exposure to the task did not produce a broad pattern of refinement toward the highest-performing strategy; instead, the dominant pattern was stability, with a more specific transition between learning-order bias groups.

Among participants who did change strategies, symptom profiles were nevertheless informative. In particular, although transitions from Other to Prototype/Exemplar occurred less often than expected by chance, the subset of individuals who made this transition showed elevated psychiatric trait scores consistent with the broader Prototype/Exemplar profile. In the transition-profile analyses, subjects who began in the Other group showed significant differences across session-two strategy outcomes, with elevated rigidity, inattention, and hyperactivity/impulsivity-related dimensions among those classified as Prototype/Exemplar in the second session. Thus, the clinical relevance of this subgroup lies not in the transition being overrepresented, but in the fact that individuals who adopted the Prototype/Exemplar strategy showed a psychiatric profile similar to those assigned to that strategy more generally.

These findings suggest that both stability and selective transitions may carry clinically meaningful information. The dominant pattern was stability in strategy membership, indicating that several strategy classes capture reproducible individual differences. At the same time, the psychiatric profiles of participants who changed strategies suggest that session-two strategy membership may still reveal meaningful latent traits. Future work should test whether these transitions reflect practice effects, state-dependent variability, or stable traits that are only expressed under repeated task exposure.

### Applications to precision psychiatry

Current psychiatric taxonomies often fail to capture clinically meaningful heterogeneity, motivating growing interest in precision phenotyping approaches. Increasingly, the field recognizes that progress in clinical assessment, intervention development, and research methodology will require a shift toward precision phenotyping (Friston et al., 2017; Anticevic and Murray, 2017; Tiego et al., 2023; Karvelis et al., 2023; Greene and Constable, 2023; Kofler et al., 2024). This shift is motivated by substantial variability within diagnoses and by the reduced statistical power inherent in grouping individuals solely according to broad diagnostic categories.

For example, individuals on the autism spectrum or with ADHD exhibit considerable variability in attention-task performance (Adamo et al., 2014; Karalunas et al., 2018; Grzadzinski et al., 2011), which influences how attentional measures are interpreted in both diagnostic and treatment contexts. Characterizing the degree to which these mechanisms differ across individuals, and how far they diverge from typical functioning, is essential for advancing more sensitive diagnostic tools and tailoring interventions to specific cognitive profiles.

A growing body of work conceptualizes neuropsychiatric symptoms as existing within a multidimensional space rather than as clustering into discrete, non-overlapping categories. Data-driven approaches, including clustering techniques applied to symptom-level data, have proven valuable for identifying patterns of natural symptom co-variation.

Identifying symptom groups that show data-driven co-variation enhances the interpretability and statistical power of modalities including neuroimaging, psychopharmacology, and genetics (Greene and Constable, 2023; Tiego et al., 2023; Ji et al., 2021). In our study, grouping individuals by distinct cognitive strategies revealed psychiatric dimensions that would likely have remained obscured under conventional diagnostic labels. This approach aligns with the aims of the Hierarchical Taxonomy of Psychopathology (Kotov et al., 2017) and the Research Domain Criteria framework (Cuthbert and Insel, 2013).

Our computational phenotyping strategy—combining remotely deployable behavioral tasks with validated self-report measures—offers a scalable and cost-effective method for reaching diverse populations while enabling integration with high-cost modalities such as neuroimaging or genotyping (Niv, 2020). The present findings represent an initial step toward refining psychiatric phenotypes and advancing precision psychiatry. Future work should extend this approach to larger clinical cohorts and pair it with clinician-rated assessments to further validate its utility in clinical settings.

### Strengths and Limitations

In this study, we used a novel Context Generalization learning task designed to distinguish diverse forms of attention and learning, previously validated through both mechanistic modeling and artificial neural network simulations. The task structure captures aspects of real-world attentional and learning demands, enhancing its interpretability for understanding everyday functioning in clinical populations and enabling the detection of psychological dimensions that may be difficult to capture with standard paradigms. Importantly, the task can be readily deployed in large clinical cohorts and paired with objective psychometric instruments or clinician-administered assessments. For example, it could be used alongside neuropsychological tools such as ADOS-2 in autism evaluations or incorporated into studies of established neurodevelopmental cohorts to identify subgroups who may benefit from more tailored interventions.

Several limitations should be noted. The sample was recruited online, and diagnostic information was based on self-report. Although many participants with ASD indicated prior formal diagnoses, independent verification was not possible within the online framework. However, the online recruitment strategy allowed us to obtain a large and diverse sample and to administer multiple psychiatric questionnaires, supporting the evaluation of a wide range of psychiatric symptoms and dimensions.

### Conclusion

We found that distinct cognitive strategies for attention, learning, and memory were strongly associated with symptoms of rigidity and inattention across ASD, ADHD, OCD, schizotypy, and psychosis. These strategies were largely stable across sessions, and individuals classified as Prototype/Exemplar in the second session showed symptom patterns similar to those who had adopted that strategy from the outset. By demonstrating that strategy-level differences in fundamental cognitive processes map onto dimensional symptom variation, these results highlight how computationally grounded behavioral measures can serve as potential precision endpoints. Such endpoints may help identify clinically meaningful cognitive subgroups and guide mechanism-informed interventions.

## Supplementary Material

1

## Figures and Tables

**Figure 2:**
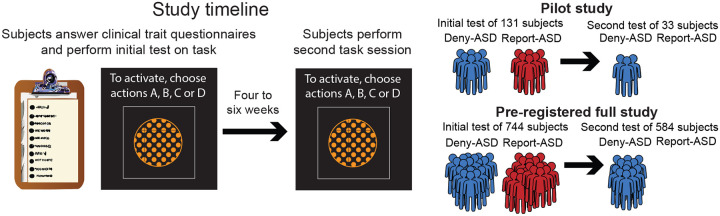
Study timeline and design The study involved subjects recruited online. They first answered a set of standardized questionnaires, then participated in two task sessions separated by 4–6 weeks. The sample was enriched for a self-reported formal diagnosis of autism spectrum disorder (ASD). Analyses were developed through a pilot and pre-registered hypotheses were tested through a full study.

**Figure 4:**
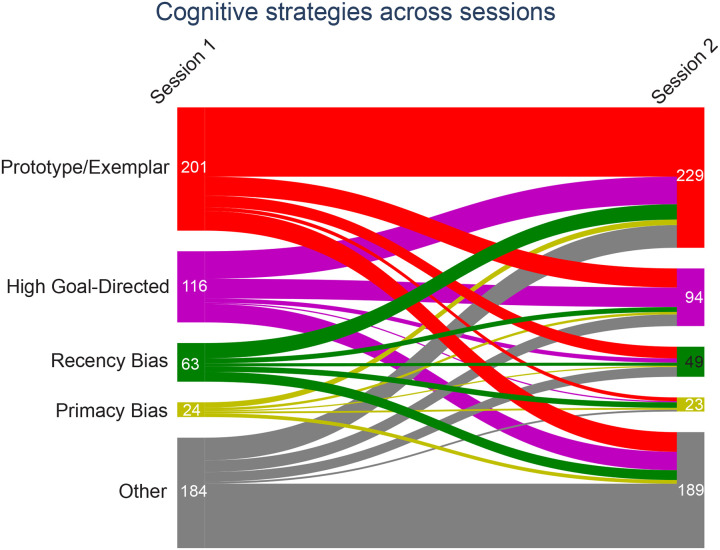
Shifts in cognitive strategies across sessions. Those subjects who completed the second session were grouped according to cognitive strategy. Subjects’ consistency or shift in cognitive strategy across the sessions was tracked.

**Figure 5:**
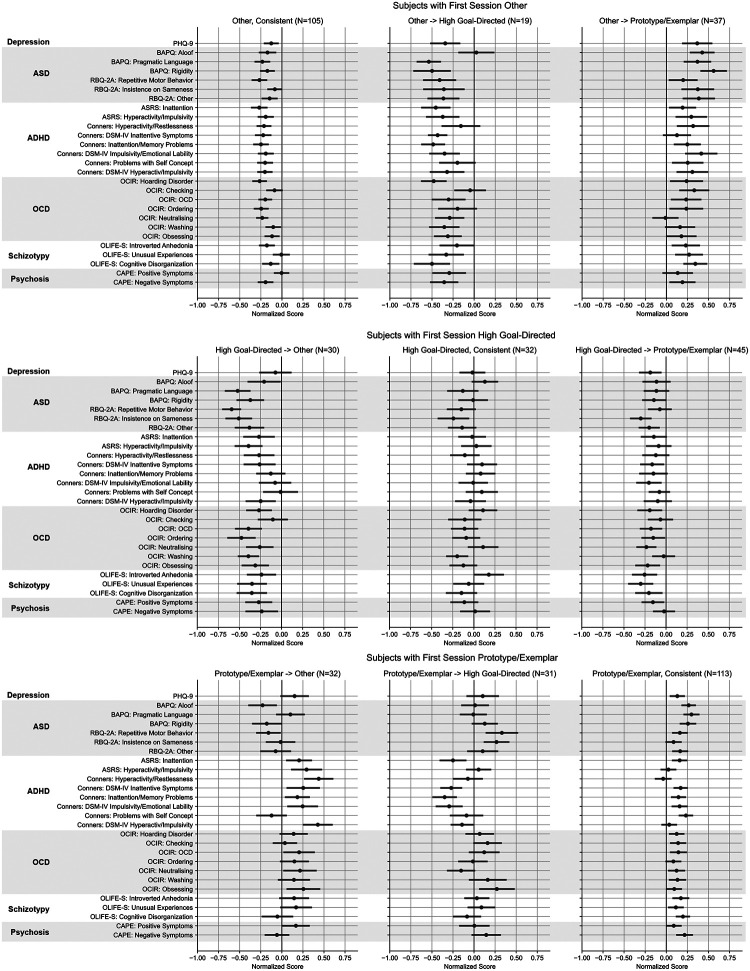
Subjects who transition between cognitive strategies across sessions exhibit unique psychiatric dimensional profiles. The psychiatric dimensional profiles were examined for subjects who exhibited the “Other,” “High Goal-Directed,” or “Prototype/Exemplar” strategy during session 1, and transitioned to any of those three cognitive strategies during session 2. Subjects who demonstrated the “Recency Bias,” or “Primacy Bias” cognitive strategies were excluded due to small sample sizes.

**Table 1:** Questionnaires.

Questionnaire	Subscale	Citation	Scoring Criteria
Patient Health Questionnaire-9 (PHQ-9)	N/A	Kroenke et al. (2001)	Kroenke et al. (2001)
Broader Autism Phenotype questionnaire (BAPQ)	Aloof Pragmatic Language Rigidity	Hurley et al. (2007)	Scored according to Hurley et al. (2007) with thresholds defined by Sasson et al. (2013)
The Adult Repetitive Behaviours Questionnaire-2 (RBQ-2a)	Repetitive Motor Behavior Insistence on Sameness Other	Barrett et al. (2015)	Barrett et al. (2015)
The World Health Organization Adult ADHD self-report scale (ASRS)	Inattention Hyperactivity/Impulsivity	Kessler et al. (2005)	Kessler et al. (2005)
Conners Rating Scales	Hyperactivity/Restlessness DSM-IV Inattentive Symptoms Inattention/Memory Problems DSM-IV Impulsivity/Emotional Lability Problems with Self Concept DSM-IV Hyperactivity/Impulsivity	Conners (2008)	Conners (2008)
Obsessive Compulsive Inventory-Revised (OCI-R)	Hoarding Disorder Checking OCD Ordering Neutralizing Washing Obsessing	Foa et al. (2002)	OCD and Hoarding Disorder scales using cutoffs described in Wootton et al. (2015), then score the OCD subscales according to cutoffs described in Abramovitch et al. (2020)
Short Scales for Measuring Schizotypy (OLIFE-S)	Introverted Anhedonia Unusual Experiences Cognitive Disorganization	Mason et al. (2005)	Mason et al. (2005)
Community Assessment of Psychotic Experiences (CAPE)	Positive Symptoms Negative Symptoms	Mossaheb et al. (2012)	Jaya et al. (2021)

## Data Availability

https://zenodo.org/records/13714285

## Code availability

https://github.com/mmint-lab/psych-disease-attention-and-learning-strategies.

## Appendix A

We preregistered several additional analyses that, for completeness, are reported here.

### Novel context generalization task version 1: early generalization versus late

We conducted preregistered multinomial logistic regression analyses of cognitive strategy group membership versus self-reported demographics (Table 1), self-reported diagnoses (Table 2), questionnaire cutoffs (Table 3), overall questionnaire scores (Table 4) and questionnaire subscales (Table 5). We found several significant results.

The demographics regression was significant for age. For the Prototype/Exemplar group, age had a significantly negative effect (−0.0060, p≤0.05), indicating that as age increases, the probability of being in the Prototype/Exemplar group decreases. For the Primacy Bias group, age had a significantly positive effect (0.0020, p≤0.05), showing that as age increases, the probability of being in the Primacy Bias group increases.

The questionnaire cutoff was significant for a positive relationship between the High Goal-Directed group and the PHQ-9 moderate cutoff (0.0795, p≤0.05).

The regression on the raw questionnaire scores was significant for the OCI-R and the RBQ-2A. For the OCI-R, the Other group had a significantly negative effect (−0.0061, p≤0.05), while the Primacy Bias group had a significantly positive effect (0.0038, p≤0.05). The RBQ-2A had a significantly positive effect on the Other group (0.1327, p≤0.05).

The questionnaire subscale regression found significant results for RBQ-2A Insistence on Sameness, BAPQ Pragmatic Language and OCI-R Hoarding Disorder. For the RBQ-2A Insistence on Sameness (IS) subscale, the High Goal-Directed group had a significantly negative effect (−0.0757, p≤0.05), while the Other group had a significantly positive effect (0.0886, p≤0.05). For the BAPQ Pragmatic Language subscale, the Other group had a significantly negative effect (−0.0692, p≤0.05), while the Prototype/Exemplar group had a significantly positive effect (0.0850, p≤0.05). For the OCI-R Hoarding Disorder subscale, the Other group had a significantly negative effect (−0.0690, p≤0.05).

It may be noted how few subscales were significant in the regression analysis relative to when they were compared individually.

When subscales were individually tested using one-way ANOVAs across strategy groups, several subscales showed nominal group differences, although only a smaller subset remained significant after Benjamini-Hochberg correction. This pattern suggests that the multinomial regression analyses may have had reduced sensitivity because of high correlation among questionnaire subscales. A well-known drawback of logistic regression analyses is when there is high correlation between variables (Daoud, 2017). That was likely the case in our logistic regression analysis.

### Individual trait questions driving separation between cognitive strategy groups

We performed LDA to identify individual questions that significantly drove the decoding of groups in session 1 (fit results in Table 6). We found that the questions generally centered on themes of inattention, hyperactivity and rigidity, even when the subscale came from a subscale with a different label (Table 8). For example, the BAPQ Pragmatic Language question “I lose track of my original point when talking to people,” also has implications for inattention. Or, the OCI-R Hoarding Disorder question “I avoid throwing things away because I am afraid I might need them later,” has additional relevance for rigidity.

Though not preregistered, we also performed LDA on the session 2 data (fit results in Table 7, significant questions in Table 9). The questions were similarly centered on traits for hyperactivity, inattention and rigidity.
