# Supplementary material for: Attention and learning strategies reveal distinct dimensions of psychiatric diseases
Source: Res Sq. 2026 Jun 23:rs.3.rs-9698354. Preprint. [Version 1] doi: 10.21203/rs.3.rs-9698354/v1 (PMC13321270; doi:10.21203/rs.3.rs-9698354/v1)
Supplement: 1 [file NIHPPRS9698354V1-supplement-1.pdf]

Supplemental Figures

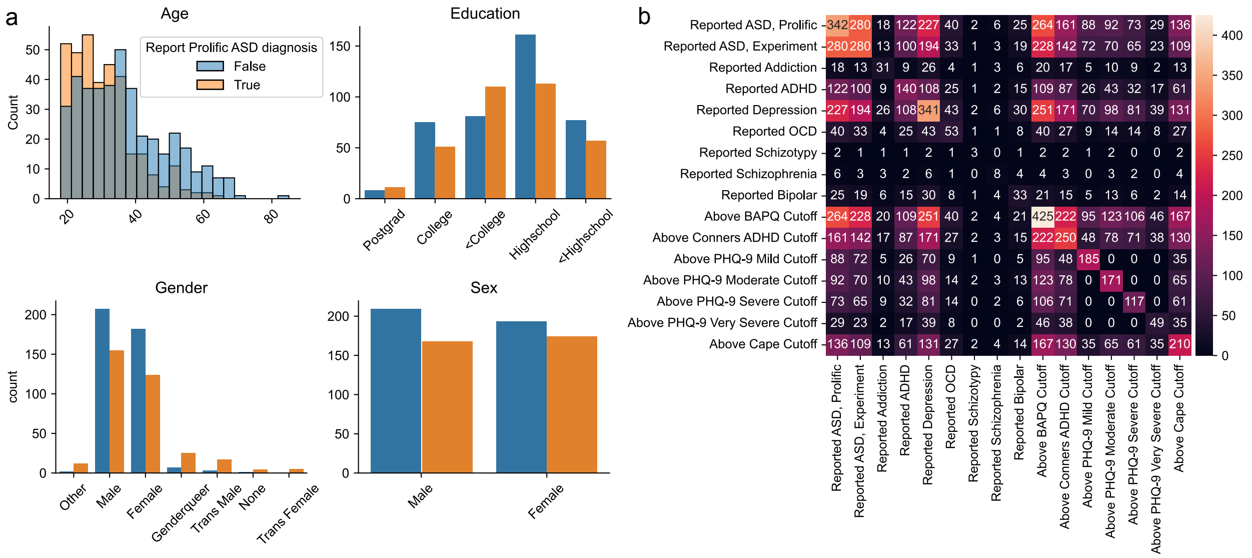

Figure S1: Subject self-reported demographics, psychiatric history and questionnaire cutoffs. (a) Subject reported demographics for age, education, gender and sex. (b) Subject counts of self-reported diagnoses, along with whether they scored above a questionnaire cutoff.

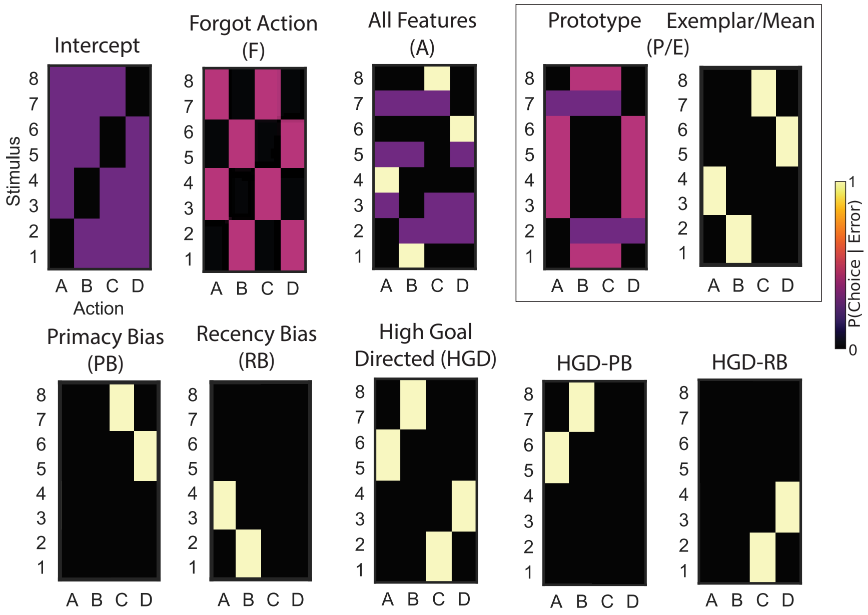

Figure S2: Attention model error pattern predictions. Each attention model predicts a distinct confusion matrix pattern of errors. During model fitting, the prototype and exemplar/mean models were both included as predictors in the same model.

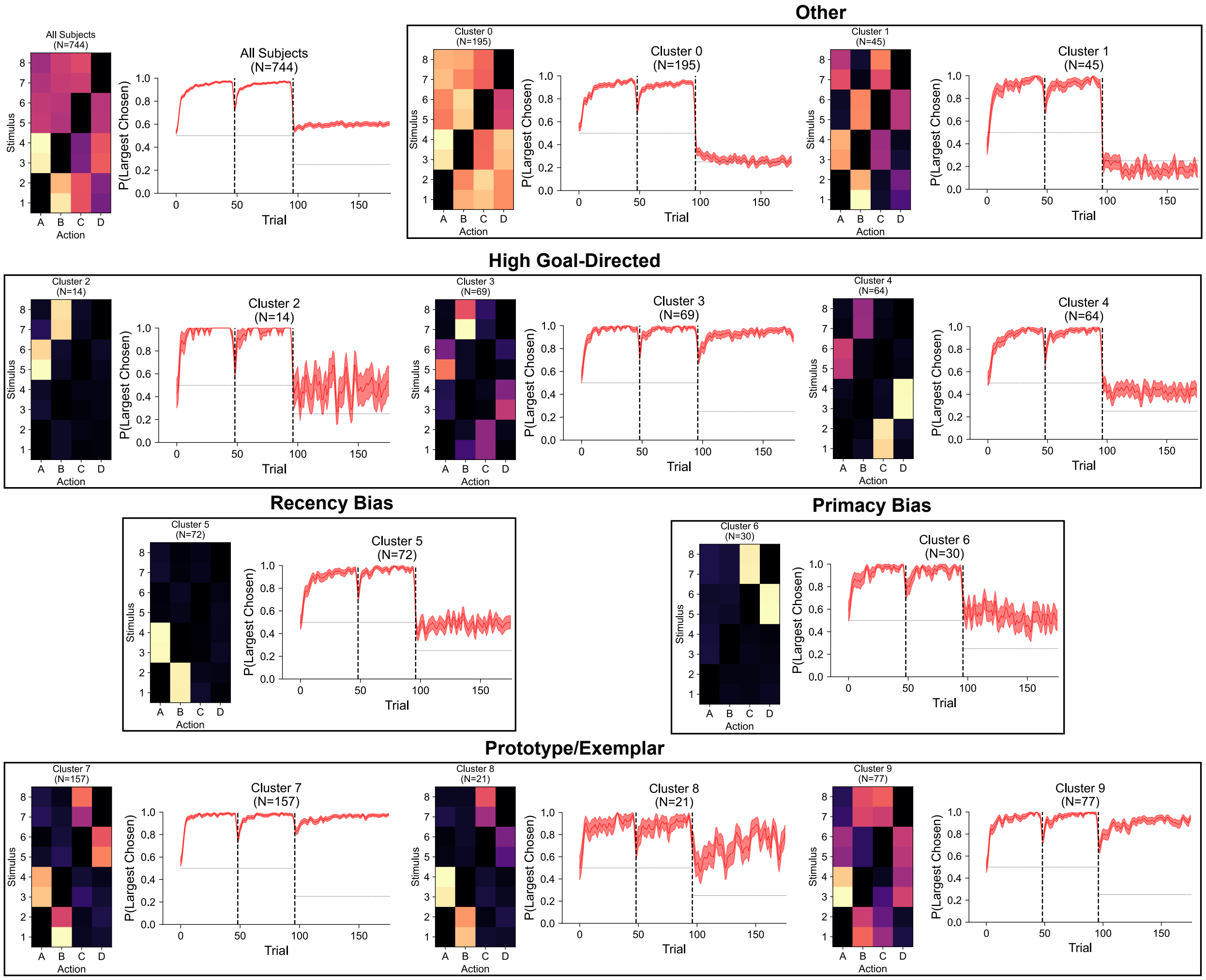

Figure S3: Cluster confusion matrices and learning curves for session 1 grouped by cognitive strategy. For each cluster, the confusion matrix and learning curves are shown. They are grouped according to the cognitive strategy they demonstrated.

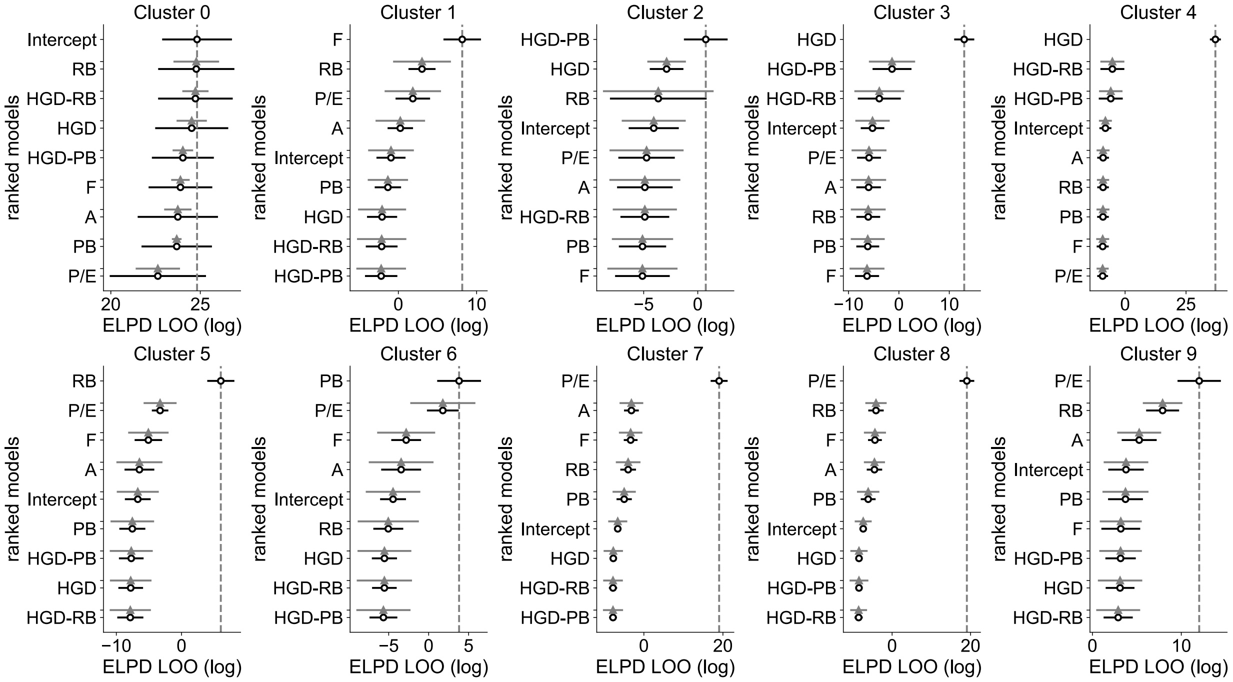

Figure S4: Cluster Bayesian model comparisons. High Goal-Directed (HGD); Prototype/Exemplar (P/E), Primacy Bias (PB); Recency Bias (RB); all-features (A); forget action (F). The attention models were fit to the confusion matrix of each cluster. Fits were compared according to leave-one-out cross-validation. The best model fit was used to identify each cluster's cognitive strategy.

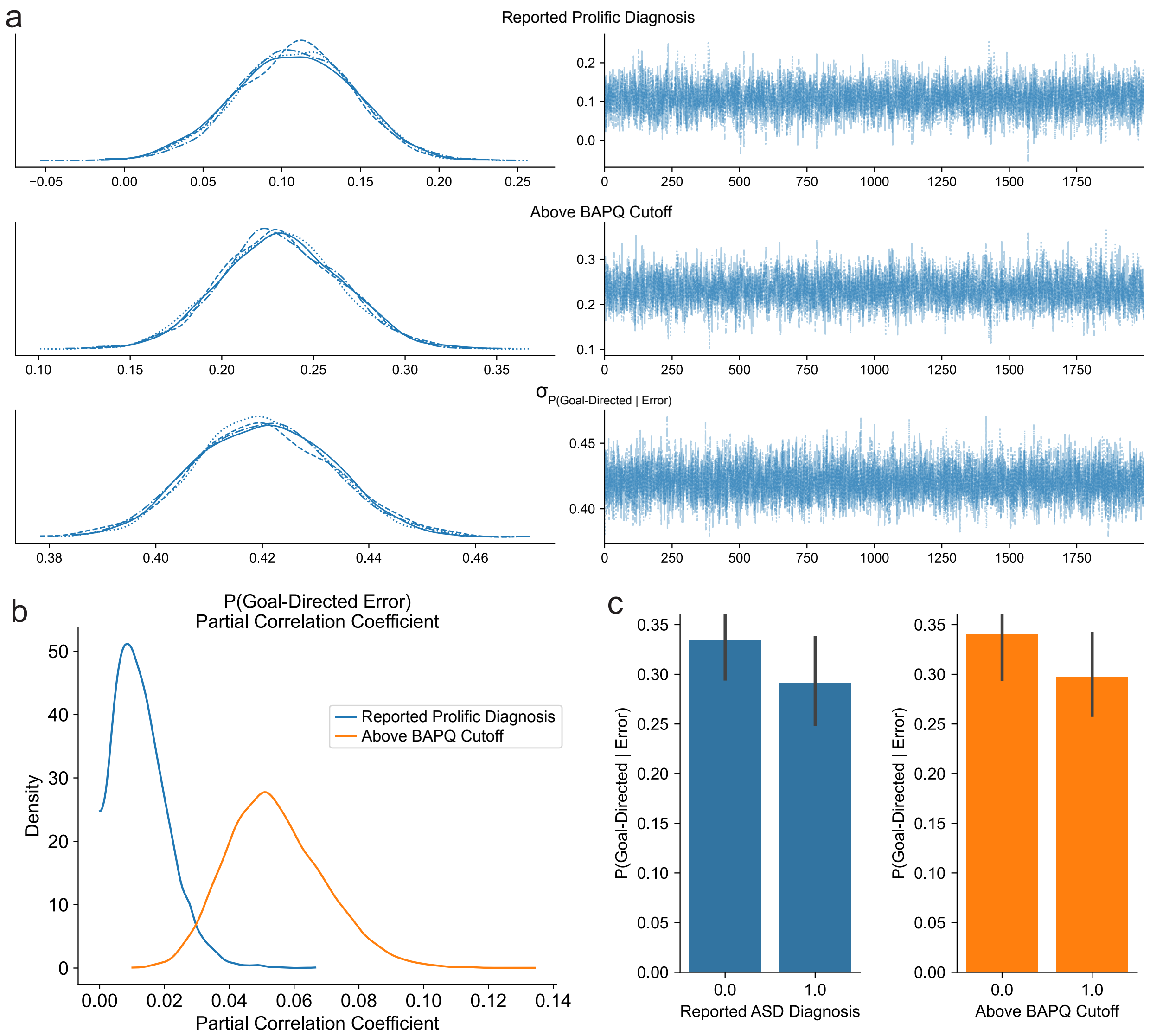

Figure S5: BAPQ cutoff is more predictive of goal-directed attention errors than self-reported ASD diagnosis. (a) Bayesian model predicted DE goal-directed attention using the reported ASD diagnosis and BAPQ cutoff for all subjects who did not display the random cognitive strategy. (b) A Bayesian model was fit to regress goal-directed attention errors using subjects' self-reported formal autism diagnosis and whether subjects scored above the BAPQ cutoff. The partial correlation coefficient for the BAPQ cutoff was significantly higher than that for reported ASD diagnosis. (c) The attention score according to reported diagnosis and BAPQ cutoff.

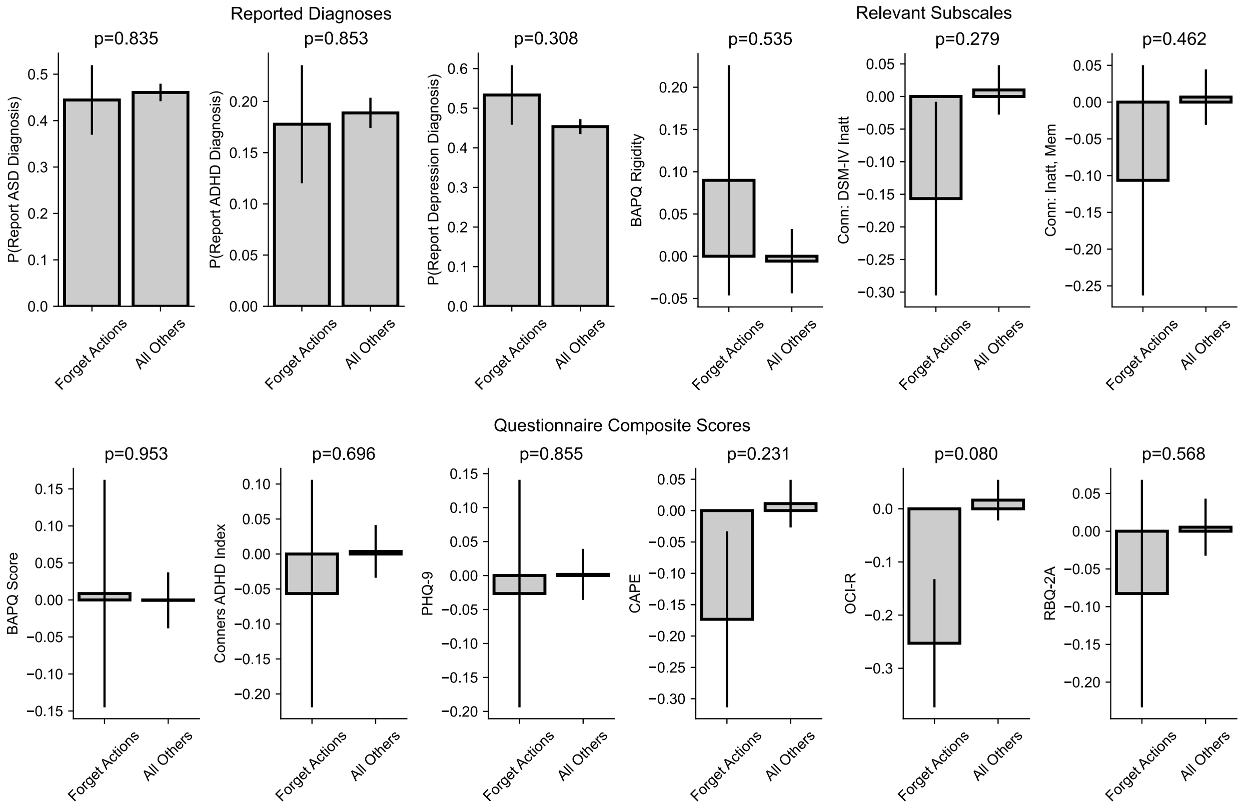

Figure S6: Psychiatric dimensional profiles of subjects who confuse actions. A subset of subjects (N=45/744) had consistent responses for stimuli within a set, but randomly associated the actions with that set. Pre-registered hypotheses did not replicate.

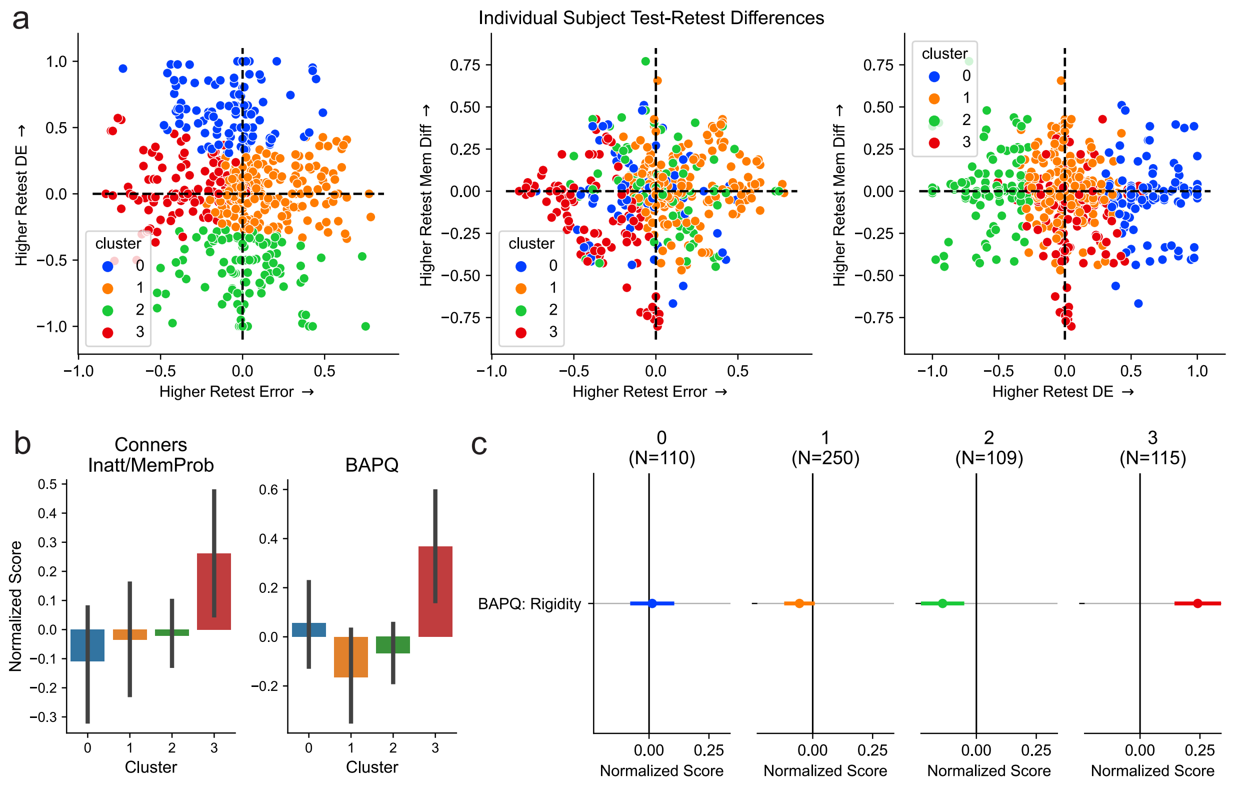

Figure S7: Shifts in attentional strategy, performance and memory across sessions are reflected by BAPQ rigidity traits using four clusters. (a) Subjects were clustered according to their shift in performance, attention and memory metrics. (b) Differences in subscale scores between clusters were tested using a one-way ANOVA with a significance threshold of $p<0.05$. There was no significant difference in the Conners Inattention/Memory Problems subscale score, but there was a significant difference in the BAPQ. (c) ANOVAs for each subscale found only BAPQ Rigidity to be significant.

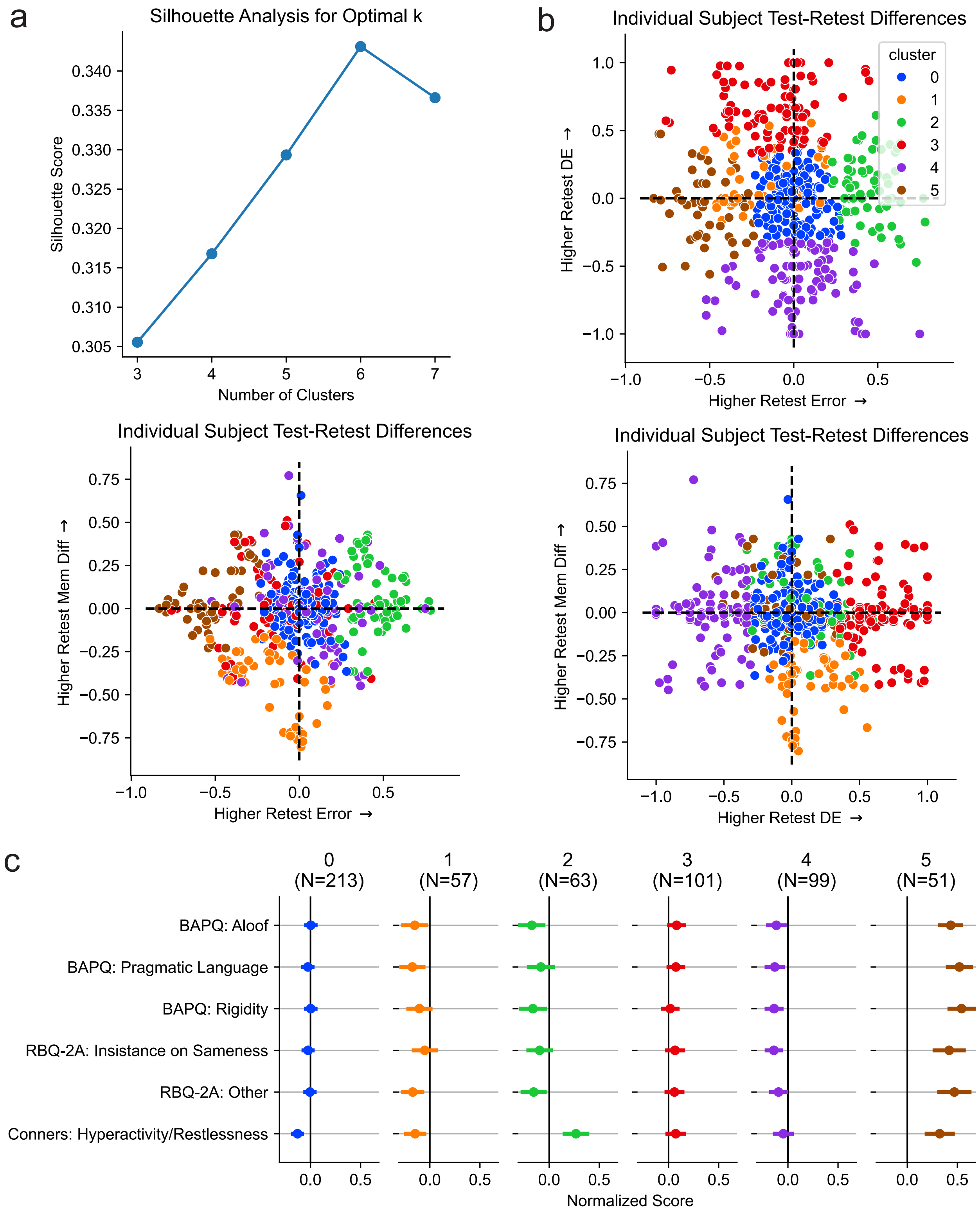

Figure S8: Shifts in attentional strategy, performance and memory across sessions are reflected by ASD and hyperactivity traits using six clusters. (a) Silhouette plot showing scores for different cluster numbers. (b) As in Fig. S7a, but with six clusters. (c) An ANOVA found that several subscale scores differed between clusters with p<0.05.

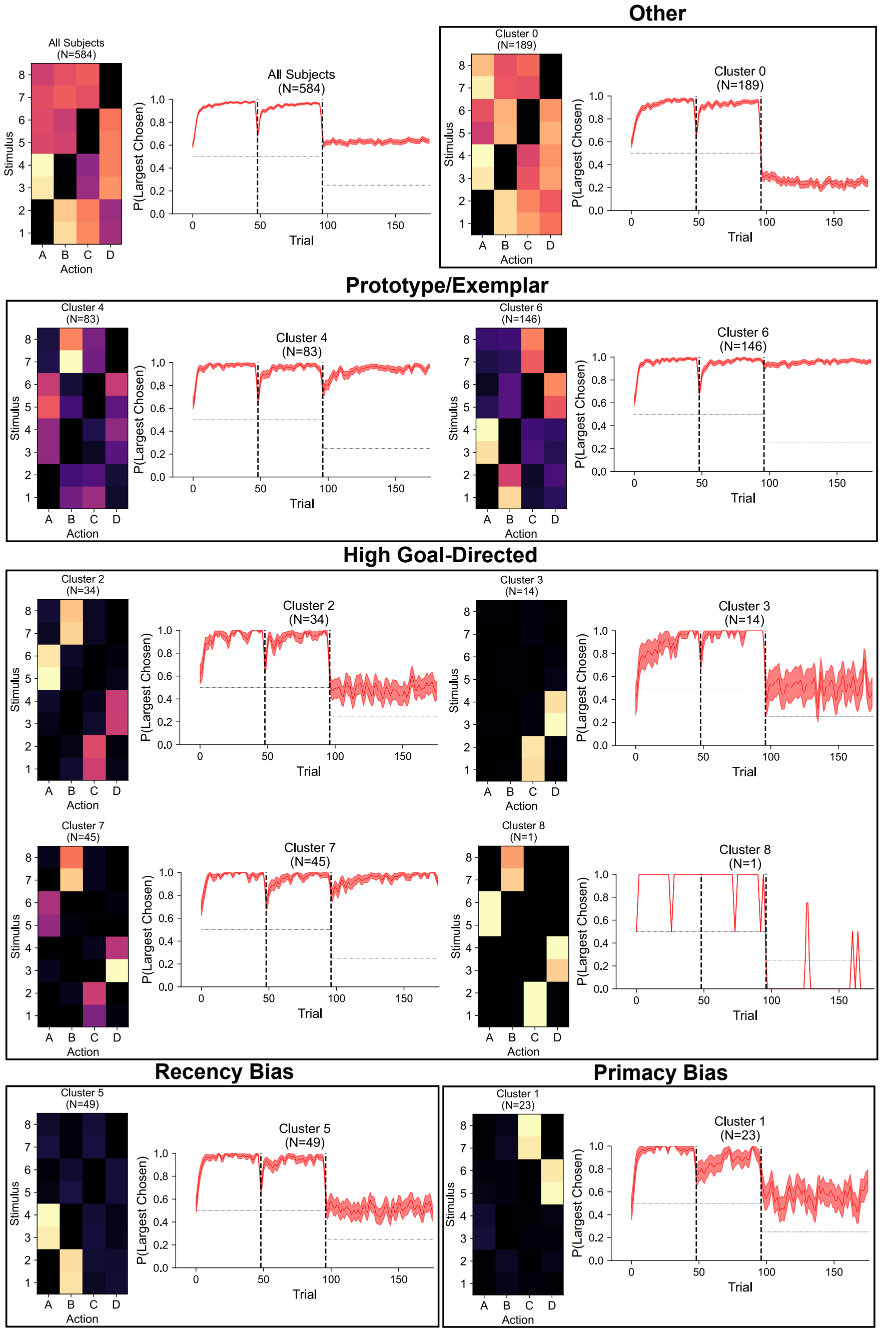

Figure S9: Cluster confusion matrices and learning curves for session 2. As in Fig. S3.

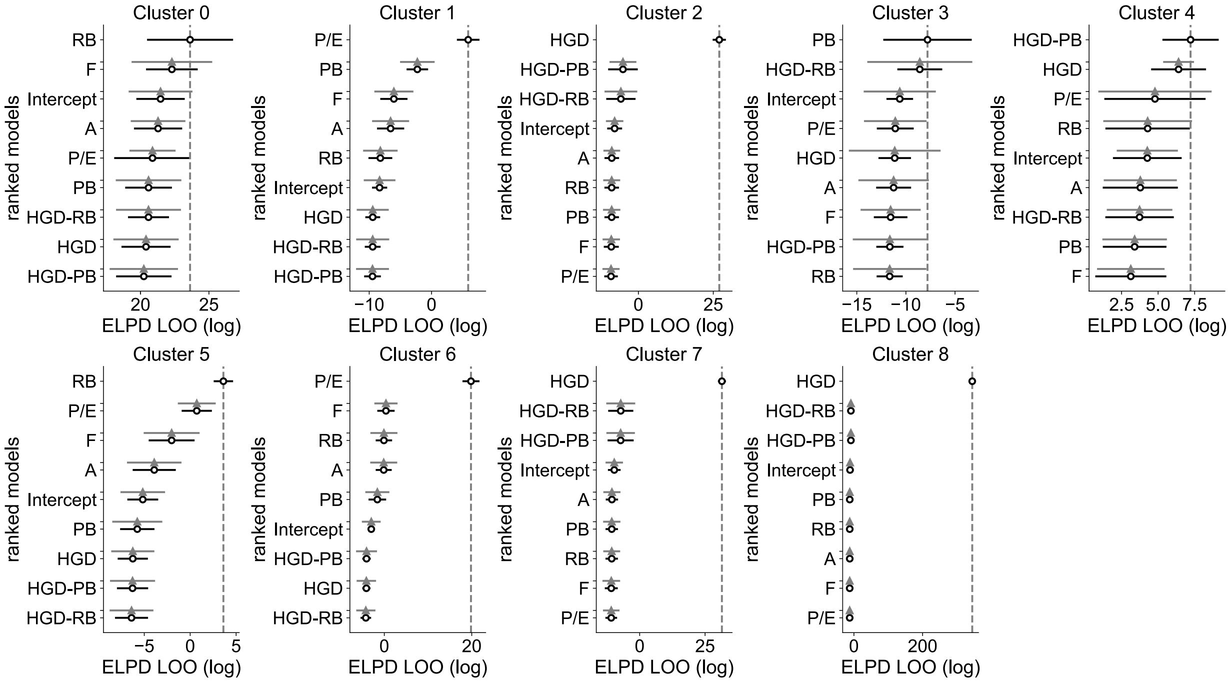

Figure S10: Cluster Bayesian model comparisons during session 2. As in Fig. S4.

Supplemental Tables

Table 1: MNLogit marginal effects on cognitive group classification for demographics

Variable	dy/dx	std err	z	P> |z|	[0.025	0.975]
**Group=High Goal-Directed**						
**C(country_of_residence)[T.1.0]**	0.2144	209.807	0.001	0.999	-411.001	411.429
**C(country_of_residence)[T.2.0]**	0.0217	0.053	0.409	0.683	-0.082	0.126
**C(country_of_residence)[T.3.0]**	0.1002	0.090	1.107	0.268	-0.077	0.277
**C(country_of_residence)[T.4.0]**	0.0605	0.046	1.323	0.186	-0.029	0.150
**C(country_of_residence)[T.5.0]**	-0.0501	0.037	-1.336	0.181	-0.123	0.023
**C(caffeine)[T.1.0]**	0.0089	0.030	0.299	0.765	-0.049	0.067
**C(sex)[T.1.0]**	-0.2258	0.191	-1.184	0.236	-0.599	0.148
**C(gender)[T.1.0]**	0.2626	0.192	1.365	0.172	-0.115	0.640
**C(gender)[T.2.0]**	0.0016	0.091	0.017	0.986	-0.177	0.180
**C(gender)[T.3.0]**	0.1006	0.087	1.159	0.247	-0.070	0.271
**C(gender)[T.4.0]**	0.2357	31600	7.45e-06	1.000	-62000	62000
**C(gender)[T.5.0]**	1.3463	331.282	0.004	0.997	-647.955	650.648
**C(gender)[T.6.0]**	-1.0607	27.825	-0.038	0.970	-55.597	53.476
**age_numeric**	0.0012	0.001	1.007	0.314	-0.001	0.004
**education**	-0.0163	0.014	-1.184	0.236	-0.043	0.011
**Group= Other**						
**C(country_of_residence)[T.1.0]**	0.2911	323.583	0.001	0.999	-633.920	634.503
**C(country_of_residence)[T.2.0]**	-0.0654	0.066	-0.984	0.325	-0.196	0.065
**C(country_of_residence)[T.3.0]**	-0.2800	0.162	-1.730	0.084	-0.597	0.037
**C(country_of_residence)[T.4.0]**	-0.1101	0.063	-1.744	0.081	-0.234	0.014
**C(country_of_residence)[T.5.0]**	0.0690	0.040	1.746	0.081	-0.008	0.147
**C(caffeine)[T.1.0]**	0.0617	0.034	1.793	0.073	-0.006	0.129
**C(sex)[T.1.0]**	0.0963	0.158	0.609	0.542	-0.213	0.406
**C(gender)[T.1.0]**	-0.1097	0.162	-0.679	0.497	-0.427	0.207
**C(gender)[T.2.0]**	-0.0179	0.098	-0.182	0.856	-0.211	0.175

Continued on next page

Variable	dy/dx	std err	z	P> |z|	[0.025	0.975]
C(gender)[T.3.0]	-0.1365	0.124	-1.100	0.271	-0.380	0.107
C(gender)[T.4.0]	0.4102	4.88e+04	8.41e-06	1.000	-9.56e+04	9.56e+04
C(gender)[T.5.0]	1.7915	514.715	0.003	0.997	-1007.032	1010.615
C(gender)[T.6.0]	0.7495	14.818	0.051	0.960	-28.293	29.792
age_numeric	0.0028	0.001	1.943	0.052	-2.46e-05	0.006
education	-0.0176	0.016	-1.086	0.278	-0.049	0.014
**Group=Prototype/Exemplar**						
C(country_of_residence)[T.1.0]	0.1580	395.375	0.000	1.000	-774.762	775.078
C(country_of_residence)[T.2.0]	0.0186	0.065	0.288	0.774	-0.108	0.146
C(country_of_residence)[T.3.0]	0.1013	0.122	0.832	0.405	-0.137	0.340
C(country_of_residence)[T.4.0]	0.0653	0.057	1.139	0.255	-0.047	0.178
C(country_of_residence)[T.5.0]	0.0057	0.042	0.138	0.891	-0.076	0.087
C(caffeine)[T.1.0]	-0.0542	0.036	-1.524	0.127	-0.124	0.015
C(sex)[T.1.0]	0.0615	0.164	0.376	0.707	-0.259	0.382
C(gender)[T.1.0]	-0.1074	0.167	-0.643	0.520	-0.435	0.220
C(gender)[T.2.0]	0.0326	0.093	0.351	0.726	-0.150	0.215
C(gender)[T.3.0]	0.0089	0.107	0.083	0.934	-0.201	0.219
C(gender)[T.4.0]	0.3619	5.96e+04	6.07e-06	1.000	-1.17e+05	1.17e+05
C(gender)[T.5.0]	-3.1682	708.095	-0.004	0.996	-1391.008	1384.672
C(gender)[T.6.0]	1.2649	15.099	0.084	0.933	-28.328	30.858
age_numeric	-0.0060	0.002	-3.769	0.000	-0.009	-0.003
education	0.0299	0.017	1.795	0.073	-0.003	0.063
**Group=Recency Bias**						
C(country_of_residence)[T.1.0]	-0.7493	1013.427	-0.001	0.999	-1987.029	1985.530
C(country_of_residence)[T.2.0]	-0.0342	0.041	-0.843	0.399	-0.114	0.045
C(country_of_residence)[T.3.0]	-0.0203	0.067	-0.302	0.762	-0.152	0.111
C(country_of_residence)[T.4.0]	0.0216	0.021	1.036	0.300	-0.019	0.062
C(country_of_residence)[T.5.0]	0.0049	0.017	0.286	0.775	-0.029	0.038

Continued on next page

Variable	dy/dx	std err	z	P> |z|	[0.025	0.975]
**C(caffeine)[T.1.0]**	-0.0299	0.018	-1.703	0.088	-0.064	0.005
**C(sex)[T.1.0]**	-0.0587	0.078	-0.752	0.452	-0.212	0.094
**C(gender)[T.1.0]**	0.0504	0.079	0.640	0.522	-0.104	0.205
**C(gender)[T.2.0]**	0.0226	0.032	0.711	0.477	-0.040	0.085
**C(gender)[T.3.0]**	0.0281	0.032	0.883	0.377	-0.034	0.090
**C(gender)[T.4.0]**	-1.1474	1.53e+05	-7.51e-06	1.000	-3e+05	3e+05
**C(gender)[T.5.0]**	-0.5348	1487.895	-0.000	1.000	-2916.755	2915.686
**C(gender)[T.6.0]**	-0.2018	9.308	-0.022	0.983	-18.444	18.041
**age_numeric**	-8.434e-05	0.001	-0.126	0.900	-0.001	0.001
**education**	0.0012	0.007	0.166	0.868	-0.013	0.015
**Group=Primacy Bias**						
**C(country_of_residence)[T.1.0]**	0.0858	84.662	0.001	0.999	-165.848	166.019
**C(country_of_residence)[T.2.0]**	0.0593	0.032	1.838	0.066	-0.004	0.123
**C(country_of_residence)[T.3.0]**	0.0989	0.053	1.855	0.064	-0.006	0.203
**C(country_of_residence)[T.4.0]**	-0.0373	0.043	-0.876	0.381	-0.121	0.046
**C(country_of_residence)[T.5.0]**	-0.0296	0.029	-1.028	0.304	-0.086	0.027
**C(caffeine)[T.1.0]**	0.0135	0.022	0.616	0.538	-0.029	0.056
**C(sex)[T.1.0]**	0.1267	0.090	1.407	0.159	-0.050	0.303
**C(gender)[T.1.0]**	-0.0958	0.093	-1.034	0.301	-0.277	0.086
**C(gender)[T.2.0]**	-0.0390	0.080	-0.489	0.625	-0.195	0.117
**C(gender)[T.3.0]**	-0.0011	0.090	-0.012	0.990	-0.177	0.175
**C(gender)[T.4.0]**	0.1396	1.28e+04	1.09e-05	1.000	-2.5e+04	2.5e+04
**C(gender)[T.5.0]**	0.5651	136.730	0.004	0.997	-267.420	268.550
**C(gender)[T.6.0]**	-0.7519	26.436	-0.028	0.977	-52.566	51.062
**age_numeric**	0.0020	0.001	2.308	0.021	0.000	0.004
**education**	0.0028	0.010	0.268	0.789	-0.017	0.023

Table 2: MNLogit marginal effects on cognitive group classification for reported diagnosis

Variable	dy/dx	std err	z	P> |z|	[0.025	0.975]
**Group=High Goal-Directed**						
**C(addiction)[T.1.0]**	-0.0338	0.0870	-0.3881	0.6980	-0.2044	0.1368
**C(adhd)[T.1.0]**	0.0421	0.0430	0.9800	0.3271	-0.0421	0.1264
**C(depression)[T.1.0]**	-0.0541	0.0329	-1.6445	0.1001	-0.1185	0.0104
**C(prolific_asd_diagnoses)[T.1.0]**	-0.0551	0.0341	-1.6187	0.1055	-0.1219	0.0116
**C(ocd)[T.1.0]**	-0.0255	0.0641	-0.3976	0.6909	-0.1511	0.1001
**C(schizophrenia)[T.1.0]**	-1.9853	373.0914	-0.0053	0.9958	-733.2310	729.2604
**Group=Other**						
**C(addiction)[T.1.0]**	0.0553	0.0854	0.6476	0.5173	-0.1120	0.2226
**C(adhd)[T.1.0]**	-0.0683	0.0485	-1.4073	0.1593	-0.1633	0.0268
**C(depression)[T.1.0]**	0.0428	0.0378	1.1309	0.2581	-0.0314	0.1169
**C(prolific_asd_diagnoses)[T.1.0]**	0.0679	0.0385	1.7661	0.0774	-0.0075	0.1433
**C(ocd)[T.1.0]**	-0.0185	0.0687	-0.2699	0.7873	-0.1532	0.1161
**C(schizophrenia)[T.1.0]**	1.4382	431.0633	0.0033	0.9973	-843.4302	846.3067
**Group=Prototype/Exemplar**						
**C(addiction)[T.1.0]**	-0.0355	0.0926	-0.3832	0.7015	-0.2170	0.1460
**C(adhd)[T.1.0]**	-0.0243	0.0493	-0.4928	0.6222	-0.1208	0.0723
**C(depression)[T.1.0]**	0.0224	0.0386	0.5797	0.5621	-0.0533	0.0981
**C(prolific_asd_diagnoses)[T.1.0]**	0.0372	0.0395	0.9415	0.3464	-0.0402	0.1146
**C(ocd)[T.1.0]**	0.0440	0.0679	0.6476	0.5172	-0.0891	0.1771
**C(schizophrenia)[T.1.0]**	1.6654	464.9605	0.0036	0.9971	-909.6403	912.9712
**Group=Recency Bias**						
**C(addiction)[T.1.0]**	0.0251	0.0265	0.9483	0.3430	-0.0268	0.0771
**C(adhd)[T.1.0]**	0.0268	0.0187	1.4385	0.1503	-0.0097	0.0634
**C(depression)[T.1.0]**	0.0283	0.0169	1.6703	0.0949	-0.0049	0.0614
**C(prolific_asd_diagnoses)[T.1.0]**	-0.0244	0.0170	-1.4368	0.1508	-0.0578	0.0089
**C(ocd)[T.1.0]**	-0.0162	0.0295	-0.5506	0.5819	-0.0741	0.0416

Continued on next page

Table 2 – continued from previous page

Variable	dy/dx	std err	z	P> |z|	[0.025	0.975]
**C(schizophrenia)[T.1.0]**	0.2060	52.1131	0.0040	0.9968	-101.9339	102.3458
**Group=Primacy Bias**						
**C(addiction)[T.1.0]**	-0.0111	0.0655	-0.1703	0.8648	-0.1394	0.1172
**C(adhd)[T.1.0]**	0.0235	0.0323	0.7282	0.4665	-0.0398	0.0869
**C(depression)[T.1.0]**	-0.0394	0.0251	-1.5673	0.1170	-0.0886	0.0099
**C(prolific_asd_diagnoses)[T.1.0]**	-0.0256	0.0257	-0.9946	0.3199	-0.0760	0.0248
**C(ocd)[T.1.0]**	0.0163	0.0443	0.3678	0.7130	-0.0705	0.1031
**C(schizophrenia)[T.1.0]**	-1.3243	1208.1964	-0.0011	0.9991	-2369.3457	2366.6970

Table 3: MNLogit marginal effects on cognitive group classification for questionnaire cutoffs

Variable	dy/dx	std err	z	P> |z|	[0.025	0.975]
**Group=High Goal-Directed**						
**C(bapq_cutoff)[T.1.0]**	-0.0365	0.0341	-1.0686	0.2853	-0.1033	0.0304
**C(conners_adhd_cutoff)[T.1.0]**	-0.0182	0.0377	-0.4826	0.6294	-0.0920	0.0556
**C(phq9_moderate_cutoff)[T.1.0]**	0.0795	0.0368	2.1628	0.0306	0.0075	0.1516
**C(phq9_severe_cutoff)[T.1.0]**	-0.0317	0.0527	-0.6020	0.5472	-0.1349	0.0715
**C(phq9_very_severe_cutoff)[T.1.0]**	0.0489	0.0719	0.6811	0.4958	-0.0919	0.1898
**C(cape_cutoff)[T.1.0]**	-0.0561	0.0383	-1.4666	0.1425	-0.1312	0.0189
**Group=Other**						
**C(bapq_cutoff)[T.1.0]**	-0.0216	0.0411	-0.5252	0.5994	-0.1021	0.0589
**C(conners_adhd_cutoff)[T.1.0]**	-0.0037	0.0439	-0.0848	0.9324	-0.0897	0.0822
**C(phq9_moderate_cutoff)[T.1.0]**	-0.0147	0.0462	-0.3170	0.7512	-0.1053	0.0759
**C(phq9_severe_cutoff)[T.1.0]**	-0.0290	0.0569	-0.5097	0.6102	-0.1406	0.0825
**C(phq9_very_severe_cutoff)[T.1.0]**	-0.0343	0.0850	-0.4034	0.6866	-0.2008	0.1322
**C(cape_cutoff)[T.1.0]**	0.0621	0.0427	1.4539	0.1460	-0.0216	0.1458
**Group=Prototype/Exemplar**						
**C(bapq_cutoff)[T.1.0]**	0.0549	0.0418	1.3138	0.1889	-0.0270	0.1369

Continued on next page

Table 3 – continued from previous page

Variable	dy/dx	std err	z	P> |z|	[0.025	0.975]
**C(conners_adhd_cutoff)[T.1.0]**	0.0029	0.0438	0.0653	0.9479	-0.0829	0.0887
**C(phq9_moderate_cutoff)[T.1.0]**	-0.0542	0.0476	-1.1380	0.2551	-0.1474	0.0391
**C(phq9_severe_cutoff)[T.1.0]**	0.0295	0.0557	0.5291	0.5967	-0.0796	0.1386
**C(phq9_very_severe_cutoff)[T.1.0]**	0.0726	0.0814	0.8928	0.3720	-0.0868	0.2321
**C(cape_cutoff)[T.1.0]**	0.0308	0.0433	0.7108	0.4772	-0.0541	0.1156
**Group=Recency Bias**						
**C(bapq_cutoff)[T.1.0]**	0.0238	0.0189	1.2614	0.2072	-0.0132	0.0608
**C(conners_adhd_cutoff)[T.1.0]**	0.0137	0.0172	0.7945	0.4269	-0.0200	0.0474
**C(phq9_moderate_cutoff)[T.1.0]**	-0.0052	0.0201	-0.2600	0.7949	-0.0447	0.0342
**C(phq9_severe_cutoff)[T.1.0]**	0.0005	0.0225	0.0200	0.9840	-0.0437	0.0446
**C(phq9_very_severe_cutoff)[T.1.0]**	0.0407	0.0253	1.6090	0.1076	-0.0089	0.0903
**C(cape_cutoff)[T.1.0]**	-0.0234	0.0190	-1.2319	0.2180	-0.0606	0.0138
**Group=Primacy Bias**						
**C(bapq_cutoff)[T.1.0]**	-0.0207	0.0258	-0.8021	0.4225	-0.0713	0.0299
**C(conners_adhd_cutoff)[T.1.0]**	0.0054	0.0280	0.1922	0.8476	-0.0494	0.0602
**C(phq9_moderate_cutoff)[T.1.0]**	-0.0055	0.0296	-0.1846	0.8535	-0.0634	0.0525
**C(phq9_severe_cutoff)[T.1.0]**	0.0308	0.0337	0.9155	0.3599	-0.0352	0.0968
**C(phq9_very_severe_cutoff)[T.1.0]**	-0.1280	0.0923	-1.3874	0.1653	-0.3089	0.0528
**C(cape_cutoff)[T.1.0]**	-0.0133	0.0281	-0.4745	0.6351	-0.0684	0.0417

Table 4: MNLogit marginal effects on cognitive group classification for questionnaire scores

Variable	dy/dx	std err	z	P> |z|	[0.025	0.975]
**Group=High Goal-Directed**						
**asrs**	0.0019	0.0026	0.7369	0.4612	-0.0032	0.0071
**bapq**	-0.0018	0.0267	-0.0684	0.9455	-0.0542	0.0505
**cape_pos_neg**	0.0102	0.0152	0.6670	0.5048	-0.0197	0.0400
**conners_full**	-0.0002	0.0011	-0.1680	0.8666	-0.0023	0.0019

Continued on next page

Variable	dy/dx	std err	z	P> |z|	[0.025	0.975]
**ocir**	0.0004	0.0020	0.2130	0.8314	-0.0035	0.0043
**olifes**	-0.0042	0.0034	-1.2183	0.2231	-0.0109	0.0025
**phq9**	0.0013	0.0034	0.3719	0.7100	-0.0054	0.0080
**rbq2a**	-0.1065	0.0579	-1.8381	0.0661	-0.2200	0.0071
**Group=Other**						
**asrs**	-0.0011	0.0031	-0.3641	0.7158	-0.0071	0.0049
**bapq**	-0.0386	0.0305	-1.2651	0.2058	-0.0985	0.0212
**cape_pos_neg**	0.0118	0.0176	0.6698	0.5030	-0.0227	0.0462
**conners_full**	-0.0008	0.0013	-0.6196	0.5355	-0.0033	0.0017
**ocir**	-0.0061	0.0023	-2.6629	0.0077	-0.0106	-0.0016
**olifes**	0.0045	0.0039	1.1516	0.2495	-0.0032	0.0122
**phq9**	0.0026	0.0040	0.6564	0.5116	-0.0052	0.0104
**rbq2a**	0.1327	0.0653	2.0343	0.0419	0.0048	0.2606
**Group=Prototype/Exemplar**						
**asrs**	-0.0014	0.0031	-0.4606	0.6451	-0.0075	0.0046
**bapq**	0.0359	0.0309	1.1623	0.2451	-0.0246	0.0964
**cape_pos_neg**	0.0020	0.0175	0.1165	0.9072	-0.0322	0.0362
**conners_full**	0.0008	0.0013	0.5946	0.5521	-0.0017	0.0032
**ocir**	0.0023	0.0022	1.0099	0.3125	-0.0021	0.0066
**olifes**	-0.0032	0.0040	-0.8079	0.4192	-0.0110	0.0046
**phq9**	-0.0015	0.0040	-0.3685	0.7125	-0.0094	0.0064
**rbq2a**	0.0676	0.0653	1.0345	0.3009	-0.0605	0.1957
**Group=Recency Bias**						
**asrs**	0.0020	0.0013	1.5007	0.1334	-0.0006	0.0045
**bapq**	0.0061	0.0134	0.4543	0.6496	-0.0201	0.0323
**cape_pos_neg**	-0.0076	0.0076	-0.9947	0.3199	-0.0225	0.0073
**conners_full**	-0.0003	0.0005	-0.5970	0.5505	-0.0013	0.0007
**ocir**	-0.0003	0.0009	-0.3695	0.7117	-0.0021	0.0015

Continued on next page

Table 4 – continued from previous page

Variable	dy/dx	std err	z	P> |z|	[0.025	0.975]
**olifes**	0.0017	0.0016	1.0196	0.3079	-0.0015	0.0048
**phq9**	0.0004	0.0016	0.2620	0.7933	-0.0027	0.0035
**rbq2a**	-0.0160	0.0270	-0.5923	0.5537	-0.0689	0.0369
**Group=Primacy Bias**						
**asrs**	-0.0014	0.0020	-0.6852	0.4932	-0.0052	0.0025
**bapq**	-0.0015	0.0195	-0.0778	0.9380	-0.0397	0.0367
**cape_pos_neg**	-0.0164	0.0123	-1.3373	0.1811	-0.0404	0.0076
**conners_full**	0.0005	0.0008	0.6395	0.5225	-0.0011	0.0021
**ocir**	0.0038	0.0014	2.6823	0.0073	0.0010	0.0065
**olifes**	0.0012	0.0025	0.4762	0.6339	-0.0037	0.0062
**phq9**	-0.0028	0.0026	-1.0667	0.2861	-0.0080	0.0024
**rbq2a**	-0.0779	0.0431	-1.8089	0.0705	-0.1623	0.0065

Table 5: MNLogit marginal effects on cognitive group classification for questionnaire subscale scores

Variable	dy/dx	std err	z	P> |z|	[0.025	0.975]
**Group=High Goal-Directed**						
**phq9_na**	-0.0130	0.0250	-0.5222	0.6015	-0.0620	0.0359
**bapq_aloof**	-0.0028	0.0241	-0.1147	0.9087	-0.0501	0.0445
**bapq_pragmatic_language**	-0.0373	0.0293	-1.2723	0.2033	-0.0946	0.0201
**bapq_rigid**	0.0058	0.0252	0.2313	0.8171	-0.0436	0.0553
**cape_pos_neg_negative_symptoms**	0.0056	0.0266	0.2089	0.8345	-0.0466	0.0577
**cape_pos_neg_positive_symptoms**	0.0134	0.0257	0.5216	0.6019	-0.0369	0.0637
**asrs_hyperactivity_impulsivity**	0.0473	0.0367	1.2878	0.1978	-0.0247	0.1193
**asrs_inattention**	-0.0037	0.0355	-0.1042	0.9170	-0.0733	0.0659
**conners_full_dsm_iv_hyperactive_impulsive_symptoms**	-0.0139	0.0386	-0.3611	0.7180	-0.0897	0.0618
**conners_full_dsm_iv_inattentive_symptoms**	0.0244	0.0363	0.6714	0.5020	-0.0468	0.0956
**conners_full_hyperactivity_restlessness**	-0.0171	0.0276	-0.6209	0.5347	-0.0712	0.0369
**conners_full_impulsivity_emotional_lability**	-0.0329	0.0292	-1.1285	0.2591	-0.0902	0.0243
**conners_full_inattention_memory_problems**	0.0203	0.0343	0.5913	0.5543	-0.0470	0.0875
**conners_full_problems_with_self_concept**	0.0302	0.0236	1.2830	0.1995	-0.0160	0.0764
**olifes_cognitive_disorganisation**	-0.0209	0.0296	-0.7066	0.4798	-0.0791	0.0372
**olifes_introvertive_anhedonia**	0.0077	0.0253	0.3035	0.7615	-0.0420	0.0574
**olifes_unusual_experiences**	-0.0375	0.0235	-1.5953	0.1106	-0.0835	0.0086
**rbq2a_is**	-0.0757	0.0314	-2.4075	0.0161	-0.1373	-0.0141

Continued on next page

Table 5 – continued from previous page

Variable	dy/dx	std err	z	P> |z|	[0.025	0.975]
**rbq2a_other**	0.0401	0.0279	1.4398	0.1499	-0.0145	0.0948
**rbq2a_rmb**	-0.0112	0.0258	-0.4324	0.6654	-0.0617	0.0394
**ocir_checking**	-0.0086	5.29e+04	-1.63e-07	1.0000	-1.04e+05	1.04e+05
**ocir_hoarding_disorder**	0.0173	0.0208	0.8338	0.4044	-0.0234	0.0580
**ocir_neutralising**	0.0151	4.62e+04	3.28e-07	1.0000	-9.06e+04	9.06e+04
**ocir_obsessing**	-0.0016	6.03e+04	-2.69e-08	1.0000	-1.18e+05	1.18e+05
**ocir_ocd**	0.0034	2.08e+05	1.62e-08	1.0000	-4.07e+05	4.07e+05
**ocir_ordering**	0.0107	5.46e+04	1.96e-07	1.0000	-1.07e+05	1.07e+05
**ocir_washing**	-0.0007	4.95e+04	-1.35e-08	1.0000	-9.71e+04	9.71e+04
**Group=Other**						
**phq9_na**	0.0280	0.0289	0.9696	0.3322	-0.0286	0.0846
**bapq_aloof**	0.0173	0.0280	0.6181	0.5365	-0.0376	0.0722
**bapq_pragmatic_language**	-0.0692	0.0337	-2.0557	0.0398	-0.1351	-0.0032
**bapq_rigid**	0.0005	0.0296	0.0164	0.9869	-0.0574	0.0584
**cape_pos_neg_negative_symptoms**	-0.0056	0.0316	-0.1765	0.8599	-0.0675	0.0563
**cape_pos_neg_positive_symptoms**	0.0345	0.0280	1.2293	0.2190	-0.0205	0.0894
**asrs_hyperactivity_impulsivity**	0.0202	0.0428	0.4728	0.6364	-0.0636	0.1041
**asrs_inattention**	-0.0171	0.0419	-0.4081	0.6832	-0.0993	0.0651
**conners_full_dsm_iv_hyperactive_impulsive_symptoms**	-0.0328	0.0443	-0.7404	0.4591	-0.1195	0.0540
**conners_full_dsm_iv_inattentive_symptoms**	-0.0181	0.0424	-0.4267	0.6696	-0.1011	0.0650

Continued on next page

Table 5 – continued from previous page

Variable	dy/dx	std err	z	P> |z|	[0.025	0.975]
**conners_full_hyperactivity_restlessness**	0.0155	0.0322	0.4834	0.6288	-0.0475	0.0786
**conners_full_impulsivity_emotional_lability**	0.0416	0.0331	1.2561	0.2091	-0.0233	0.1066
**conners_full_inattention_memory_problems**	-0.0164	0.0402	-0.4084	0.6830	-0.0951	0.0623
**conners_full_problems_with_self_concept**	-0.0238	0.0275	-0.8674	0.3857	-0.0777	0.0300
**olifes_cognitive_disorganisation**	0.0637	0.0337	1.8875	0.0591	-0.0024	0.1298
**olifes_introvertive_anhedonia**	-0.0254	0.0295	-0.8591	0.3903	-0.0833	0.0325
**olifes_unusual_experiences**	0.0094	0.0265	0.3555	0.7222	-0.0425	0.0614
**rbq2a_is**	0.0886	0.0350	2.5279	0.0115	0.0199	0.1573
**rbq2a_other**	0.0286	0.0309	0.9242	0.3554	-0.0320	0.0891
**rbq2a_rmb**	-0.0278	0.0291	-0.9551	0.3395	-0.0849	0.0293
**ocir_checking**	0.0453	nan	nan	nan	nan	nan
**ocir_hoarding_disorder**	-0.0690	0.0237	-2.9102	0.0036	-0.1154	-0.0225
**ocir_neutralising**	-0.0544	nan	nan	nan	nan	nan
**ocir_obsessing**	0.0006	nan	nan	nan	nan	nan
**ocir_ocd**	-0.0151	nan	nan	nan	nan	nan
**ocir_ordering**	-0.0311	nan	nan	nan	nan	nan
**ocir_washing**	-0.0274	nan	nan	nan	nan	nan
**Group=Prototype/Exemplar**						
**phq9_na**	6.40e-05	0.0292	0.0022	0.9983	-0.0573	0.0574
**bapq_aloof**	-0.0201	0.0292	-0.6880	0.4915	-0.0773	0.0371

Continued on next page

Table 5 – continued from previous page

Variable	dy/dx	std err	z	P> |z|	[0.025	0.975]
**bapq_pragmatic_language**	0.0850	0.0342	2.4847	0.0130	0.0180	0.1521
**bapq_rigid**	0.0202	0.0305	0.6649	0.5061	-0.0394	0.0799
**cape_pos_neg_negative_symptoms**	0.0226	0.0311	0.7256	0.4681	-0.0384	0.0836
**cape_pos_neg_positive_symptoms**	-0.0229	0.0283	-0.8095	0.4182	-0.0784	0.0326
**asrs_hyperactivity_impulsivity**	-0.0631	0.0433	-1.4587	0.1446	-0.1480	0.0217
**asrs_inattention**	0.0070	0.0423	0.1646	0.8692	-0.0759	0.0898
**conners_full_dsm_iv_hyperactive_impulsive_symptoms**	0.0343	0.0445	0.7705	0.4410	-0.0529	0.1214
**conners_full_dsm_iv_inattentive_symptoms**	0.0243	0.0429	0.5669	0.5708	-0.0597	0.1083
**conners_full_hyperactivity_restlessness**	0.0150	0.0325	0.4629	0.6435	-0.0487	0.0788
**conners_full_impulsivity_emotional_lability**	-0.0140	0.0340	-0.4124	0.6800	-0.0806	0.0526
**conners_full_inattention_memory_problems**	-0.0308	0.0404	-0.7637	0.4450	-0.1099	0.0483
**conners_full_problems_with_self_concept**	-0.0142	0.0284	-0.5016	0.6159	-0.0698	0.0414
**olifes_cognitive_disorganisation**	-0.0460	0.0347	-1.3250	0.1852	-0.1140	0.0220
**olifes_introvertive_anhedonia**	0.0134	0.0303	0.4435	0.6574	-0.0459	0.0728
**olifes_unusual_experiences**	0.0126	0.0269	0.4698	0.6385	-0.0401	0.0654
**rbq2a_is**	-0.0067	0.0362	-0.1845	0.8536	-0.0777	0.0643
**rbq2a_other**	-0.0214	0.0312	-0.6836	0.4942	-0.0826	0.0399
**rbq2a_rmb**	0.0357	0.0290	1.2292	0.2190	-0.0212	0.0926
**ocir_checking**	-0.0084	1.67e+05	-5.00e-08	1.0000	-3.28e+05	3.28e+05
**ocir_hoarding_disorder**	0.0194	0.0238	0.8125	0.4165	-0.0273	0.0660

Continued on next page

Table 5 – continued from previous page

Variable	dy/dx	std err	z	P> |z|	[0.025	0.975]
**ocir_neutralising**	0.0255	1.46e+05	1.74e-07	1.0000	-2.87e+05	2.87e+05
**ocir_obsessing**	-0.0047	1.91e+05	-2.44e-08	1.0000	-3.74e+05	3.74e+05
**ocir_ocd**	0.0049	6.58e+05	7.41e-09	1.0000	-1.29e+06	1.29e+06
**ocir_ordering**	-0.0179	1.73e+05	-1.03e-07	1.0000	-3.39e+05	3.39e+05
**ocir_washing**	0.0310	1.57e+05	1.98e-07	1.0000	-3.07e+05	3.07e+05
**Group=Recency Bias**						
**phq9_na**	-0.0017	0.0115	-0.1458	0.8841	-0.0243	0.0209
**bapq_aloof**	0.0057	0.0128	0.4488	0.6536	-0.0193	0.0307
**bapq_pragmatic_language**	0.0154	0.0154	0.9987	0.3179	-0.0148	0.0455
**bapq_rigid**	-0.0248	0.0136	-1.8269	0.0677	-0.0515	0.0018
**cape_pos_neg_negative_symptoms**	-0.0172	0.0133	-1.2941	0.1956	-0.0433	0.0089
**cape_pos_neg_positive_symptoms**	0.0008	0.0121	0.0698	0.9444	-0.0229	0.0246
**asrs_hyperactivity_impulsivity**	0.0127	0.0177	0.7188	0.4723	-0.0219	0.0474
**asrs_inattention**	0.0269	0.0179	1.5040	0.1326	-0.0082	0.0619
**conners_full_dsm_iv_hyperactive_impulsive_symptoms**	-0.0127	0.0186	-0.6800	0.4965	-0.0492	0.0238
**conners_full_dsm_iv_inattentive_symptoms**	-0.0058	0.0175	-0.3336	0.7387	-0.0402	0.0285
**conners_full_hyperactivity_restlessness**	0.0031	0.0128	0.2456	0.8060	-0.0219	0.0282
**conners_full_impulsivity_emotional_lability**	0.0013	0.0134	0.0965	0.9231	-0.0250	0.0276
**conners_full_inattention_memory_problems**	-0.0100	0.0164	-0.6127	0.5401	-0.0421	0.0221
**conners_full_problems_with_self_concept**	0.0065	0.0116	0.5580	0.5768	-0.0163	0.0293

Continued on next page

Table 5 – continued from previous page

Variable	dy/dx	std err	z	P> |z|	[0.025	0.975]
**olifes_cognitive_disorganisation**	-0.0019	0.0148	-0.1285	0.8977	-0.0309	0.0271
**olifes_introvertive_anhedonia**	0.0181	0.0127	1.4183	0.1561	-0.0069	0.0431
**olifes_unusual_experiences**	-0.0043	0.0108	-0.3941	0.6935	-0.0255	0.0170
**rbq2a_is**	0.0047	0.0149	0.3133	0.7540	-0.0246	0.0339
**rbq2a_other**	-0.0153	0.0129	-1.1844	0.2363	-0.0405	0.0100
**rbq2a_rmb**	0.0081	0.0118	0.6877	0.4916	-0.0150	0.0313
**ocir_checking**	-0.0249	7.26e+04	-3.44e-07	1.0000	-1.42e+05	1.42e+05
**ocir_hoarding_disorder**	0.0145	0.0094	1.5417	0.1232	-0.0039	0.0330
**ocir_neutralising**	-0.0098	6.34e+04	-1.54e-07	1.0000	-1.24e+05	1.24e+05
**ocir_obsessing**	0.0057	8.27e+04	6.92e-08	1.0000	-1.62e+05	1.62e+05
**ocir_ocd**	-0.0030	2.85e+05	-1.04e-08	1.0000	-5.59e+05	5.59e+05
**ocir_ordering**	0.0080	7.48e+04	1.07e-07	1.0000	-1.47e+05	1.47e+05
**ocir_washing**	0.0075	6.80e+04	1.10e-07	1.0000	-1.33e+05	1.33e+05
**Group=Primacy Bias**						
**phq9_na**	-0.0134	0.0190	-0.7023	0.4825	-0.0506	0.0239
**bapq_aloof**	-0.0002	0.0185	-0.0092	0.9926	-0.0363	0.0360
**bapq_pragmatic_language**	0.0060	0.0222	0.2719	0.7857	-0.0375	0.0496
**bapq_rigid**	-0.0017	0.0195	-0.0887	0.9293	-0.0400	0.0365
**cape_pos_neg_negative_symptoms**	-0.0054	0.0208	-0.2570	0.7972	-0.0462	0.0355
**cape_pos_neg_positive_symptoms**	-0.0258	0.0204	-1.2640	0.2062	-0.0659	0.0142

Continued on next page

Table 5 – continued from previous page

Variable	dy/dx	std err	z	P> |z|	[0.025	0.975]
**asrs_hyperactivity_impulsivity**	-0.0171	0.0278	-0.6153	0.5383	-0.0716	0.0374
**asrs_inattention**	-0.0130	0.0265	-0.4923	0.6225	-0.0650	0.0389
**conners_full_dsm_iv_hyperactive_impulsive_symptoms**	0.0251	0.0292	0.8594	0.3901	-0.0322	0.0825
**conners_full_dsm_iv_inattentive_symptoms**	-0.0248	0.0275	-0.8988	0.3688	-0.0787	0.0292
**conners_full_hyperactivity_restlessness**	-0.0166	0.0212	-0.7825	0.4339	-0.0582	0.0250
**conners_full_impulsivity_emotional_lability**	0.0040	0.0215	0.1878	0.8510	-0.0381	0.0461
**conners_full_inattention_memory_problems**	0.0370	0.0258	1.4314	0.1523	-0.0137	0.0876
**conners_full_problems_with_self_concept**	0.0013	0.0175	0.0758	0.9396	-0.0330	0.0357
**olifes_cognitive_disorganisation**	0.0052	0.0221	0.2346	0.8145	-0.0380	0.0484
**olifes_introvertive_anhedonia**	-0.0138	0.0195	-0.7094	0.4781	-0.0520	0.0244
**olifes_unusual_experiences**	0.0197	0.0170	1.1586	0.2466	-0.0136	0.0529
**rbq2a_is**	-0.0109	0.0228	-0.4779	0.6327	-0.0556	0.0338
**rbq2a_other**	-0.0321	0.0207	-1.5498	0.1212	-0.0726	0.0085
**rbq2a_rmb**	-0.0049	0.0193	-0.2510	0.8018	-0.0427	0.0330
**ocir_checking**	-0.0034	2.52e+05	-1.34e-08	1.0000	-4.94e+05	4.94e+05
**ocir_hoarding_disorder**	0.0178	0.0150	1.1830	0.2368	-0.0117	0.0472
**ocir_neutralising**	0.0236	2.20e+05	1.07e-07	1.0000	-4.32e+05	4.32e+05
**ocir_obsessing**	-7.18e-05	2.87e+05	-2.50e-10	1.0000	-5.63e+05	5.63e+05
**ocir_ocd**	0.0098	9.91e+05	9.92e-09	1.0000	-1.94e+06	1.94e+06
**ocir_ordering**	0.0303	2.60e+05	1.16e-07	1.0000	-5.10e+05	5.10e+05

Continued on next page

Table 5 – continued from previous page

**Variable**	**dy/dx**	**std err**	**z**	**P> |z|**	**[0.025**	**0.975]**
**ocir_washing**	-0.0104	2.36e+05	-4.42e-08	1.0000	-4.63e+05	4.63e+05

Table 6: LDA Statistics for Session 1

Statistic	Value	Split	Label
Mean	0.372083	Train	Control
SEM	0.003471	Train	Control
Mean	0.372083	Test	Control
SEM	0.003471	Test	Control
Mean	0.406379	Train	Sample
SEM	0.000071	Train	Sample
Mean	0.369624	Test	Sample
SEM	0.017709	Test	Sample
P(Sample¿Shuffled)	1.000000	Train	Comparison
P(Sample¿Shuffled)	1.000000	Test	Comparison

Table 7: LDA Statistics for Session 2

Statistic	Value	Split	Label
Mean	0.462178	Train	Control
SEM	0.004645	Train	Control
Mean	0.462178	Test	Control
SEM	0.004645	Test	Control
Mean	0.481825	Train	Sample
SEM	0.000145	Train	Sample
Mean	0.386986	Test	Sample
SEM	0.020172	Test	Sample
P(Sample¿Shuffled)	1.000000	Train	Comparison
P(Sample¿Shuffled)	1.000000	Test	Comparison

Table 8: Top LDA Questions for Session 1

questionnaire	subscale	question	weights
CAPE	Positive Symptoms	Do you ever see objects, people or animals that other people cannot see?	0.392826
OLIFE-S	Introvertive Anhedonia	Is trying new foods something you have always enjoyed?	0.357712
OCIR	Hoarding Disorder	I avoid throwing things away because I am afraid I might need them later.	0.235053
CAPE	Positive Symptoms	Do you ever feel as if people seem to drop hints about you or say things with a double meaning?	0.224813
Conners	Hyperactivity Restlessness	I tend to squirm or fidget.	0.192770
BAPQ	Pragmatic Language	I can tell when someone is not interested in what I am saying***.	0.191519
OLIFE-S	Cognitive Disorganisation	Is it hard for you to make decisions?	0.159883
ASRS	Inattention	How often do you misplace or have difficulty finding things at home or at work?	0.156819
BAPQ	Pragmatic Language	I can tell when it is time to change topics in conversation***.	0.131317
BAPQ	Pragmatic Language	I lose track of my original point when talking to people.	0.107854
RBQ-2A	RMB	Do you repetitively fiddle with items (e.g. spin, twiddle, bang, tap, twist, or flick anything repeatedly)?	0.078782
ASRS	Hyperactivity-Impulsivity	How often do you fidget or squirm with your hands or your feet when you have to sit down for a long time?	0.006833

Table 9: Top LDA Questions for Session 2

questionnaire	subscale	question	weights
ASRS	Hyperactivity-Impulsivity	How often do you fidget or squirm with your hands or your feet when you have to sit down for a long time?	0.644058
OLIFE-S	Unusual Experiences	Have you sometimes sensed an evil presence around you, even though you could not see it?	0.596468
Conners	DSM-IV Hyperactive-Impulsive Symptoms	I fidget (with my hands or feet) or squirm in my seat.	0.402952
RBQ-2A	RMB	Do you rock backwards and forwards, or side to side, either when sitting or when standing?	0.353852
OCIR	Ordering, OCD	I get upset if others change the way I have arranged things.	0.324864
CAPE	Negative Symptoms	Do you ever feel that you are not much of a talker when you are conversing with other people?	0.276999
Conners	Problems With Self-Concept	I'm not sure of myself.	0.254647
CAPE	Negative Symptoms	Do you ever feel that you are neglecting your appearance or personal hygiene?	0.243612
BAPQ	Aloof	I enjoy chatting with people***.	0.230985
BAPQ	Aloof	Conversation bores me***.	0.227434
BAPQ	Pragmatic Language	I am "in-tune" with the other person during conversation***.	0.216593
BAPQ	Pragmatic Language	I feel disconnected or "out of sync" in conversations with others***.	0.212698
BAPQ	Aloof	I am good at making small talk***.	0.206245
BAPQ	Aloof	When I make conversation it is just to be polite***.	0.183448
BAPQ	Rigid	People have to talk me into trying something new.	0.175917
BAPQ	Rigid	I have to warm myself up to the idea of visiting an unfamiliar place.	0.166782
BAPQ	Pragmatic Language	I leave long pauses in conversation.	0.157519
ASRS	Hyperactivity-Impulsivity	How often do you feel restless or fidgety?	0.156749
BAPQ	Rigid	I alter my daily routine by trying something different.	0.144148
Conners	Hyperactivity Restlessness	I tend to squirm or fidget.	0.143492
BAPQ	Pragmatic Language	I find it hard to get my words out smoothly	0.129695
BAPQ	Rigid	I act very set in my ways.	0.118802

Conners	Problems With Self-Concept, ADHD Index	I avoid new challenges because I lack faith in my abilities.	0.115916
OLIFE-S	Cognitive Disorganisation	Is it hard for you to make decisions?	0.111402
BAPQ	Rigid	People get frustrated by my unwillingness to bend.	0.108430
RBQ-2A	RMB	Do you make repetitive hand and/or finger movements (e.g. flap, wave, or flick your hands or fingers repetitively)?	0.106494
BAPQ	Aloof	I would rather talk to people to get information than to socialize.	0.084474
CAPE	Negative Symptoms	Do you ever feel that you can never get things done?	0.077279
Conners	Problems With Self-Concept	I wish I had greater confidence in my abilities.	0.072932
BAPQ	Aloof	I look forward to situations where I can meet new people.	0.069989
Conners	ADHD Index	Sometimes my attention narrows so much that I'm oblivious to everything else; other times it's so broad that everything distracts me.	0.065258
OLIFE-S	Cognitive Disorganisation	Are you easily distracted when you read or talk to someone?	0.060715
RBQ-2A	Other	If you are left to occupy yourself, will you choose from a restricted range of repetitive activities?	0.055764
RBQ-2A	RMB	Do you repetitively fiddle with items (e.g. spin, twiddle, bang, tap, twist, or flick anything repeatedly)?	0.051382
BAPQ	Pragmatic Language	My voice has a flat or monotone sound to it.	0.050717
BAPQ	Pragmatic Language	People ask me to repeat things I've said because they don't understand.	0.048383
ASRS	Inattention	How often do you have difficulty getting things in order when you have to do a task that requires organization?	0.032438
ASRS	Inattention	When you have a task that requires a lot of thought, how often do you avoid or delay getting started?	0.020108
Conners	Inattention/Memory Problems, ADHD Index	I can't get things done unless there's an absolute deadline.	0.009004
